# RNA elements and their biotechnological applications in plants

**DOI:** 10.1111/nph.70400

**Published:** 2025-07-27

**Authors:** Filip Lastovka, Hadrien Peyret, George P. Lomonossoff, Betty Y.‐W. Chung

**Affiliations:** ^1^ Department of Pathology University of Cambridge Tennis Court Road Cambridge CB2 1QP UK; ^2^ Department of Biochemistry and Metabolism John Innes Centre Norwich Research Park Norwich NR4 7UH UK; ^3^ Division of Plant and Crop Sciences, Sutton Bonnington campus University of Nottingham Sutton Bonington Loughborough LE12 5RD UK

**Keywords:** biotechnology, gene expression, plant, RNA element, RNA turnover, transcription, translation

## Abstract

Engineering of plants for improved traits and efficient heterologous protein production can be achieved by modifying or introducing *cis*‐ or *trans*‐acting RNA elements. The function of these elements depends not only on their nucleotide sequence but also on their highly dynamic higher order structures. In this review, we explore RNA regulatory elements with established or potential application in plant biotechnology. We discuss RNA elements involved in translational control, transcript stability, and protein coproduction, as well as RNA domains that mediate conditional expression, RNA decay, or cap‐independent initiation. While some of these elements can be used in transiently or stably transformed plants, others have proven valuable in plant‐based *in vitro* expression systems. Additionally, we highlight RNA elements important for plastid gene expression. Finally, we examine RNA elements that are yet to be applied in plant biotechnology but have been successfully used in other organisms or require further understanding before they can be effectively utilized.

**Table:** 

	Contents	
	Summary	2517
I.	Introduction	2517
II.	Translational control as a tool for fine‐tuning gene expression	2519
III.	Functional RNA domains	2521
IV.	Other RNA elements	2526
V.	RNA elements in engineered chloroplasts	2529
VI.	Perspectives	2532
	Acknowledgements	2532
	References	2532

## I. Introduction

Genetic engineering techniques have allowed us to cultivate plants with improved properties or use them as expression systems for synthesis of heterologous proteins. To achieve desired plant qualities or ensure effective protein production, it is important to control the levels of expression both spatially and temporally. Key tools to achieve the desired gene expression levels in the modified plants are *cis*‐ or *trans*‐acting RNA regulatory elements, which can be modified in the endogenous genome or introduced with heterologous sequences.

**Fig. 1:**
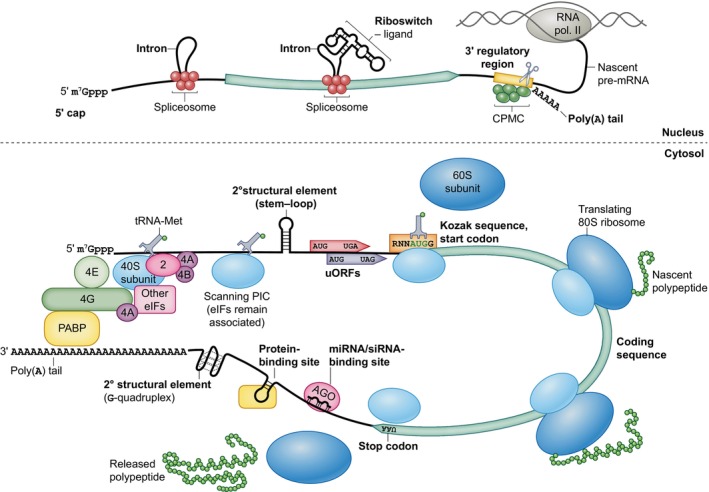
Processing and translation of nucleus‐derived mRNA in eukaryotes. RNA elements are highlighted in bold. eIFs, eukaryotic translation initiation factors. Specific translation initiation factors are illustrated individually and numbered. PIC, preinitiation complex, which consists of the 40S ribosomal subunit, eIF1, eIF1A, eIF3, eIF5, and a ternary complex of methionyl initiator tRNA, eIF2, and GTP; the PIC scans for a start codon in association with the cap‐binding complex: eIF4A, eIF4E/eIFiso4E, and eIF4G/eIFiso4G (Browning *et al*., 1992; Jackson *et al*., 2010; Brito Querido *et al*., 2024). The initiation factors remain associated with the small ribosomal subunit until start codon recognition and recruitment of the 60S ribosomal subunit but are omitted from the illustration for simplicity. Our knowledge of the eukaryotic translation machinery mostly comes from yeast and mammalian cells. Study of plant translation has revealed and will continue to reveal that the machinery differs in multiple ways both in its composition and regulation, for example through the use of the plant‐specific alternative initiation factors eIFiso4E and eIFiso4G (Browning, 2014). AGO, Argonaute; CPMC, cleavage and polyadenylation molecular complex; PABP, poly(A)‐binding protein; uORF, upstream open reading frame. The illustrated RNA elements are not present on all mRNAs.

Similarly to other eukaryotes, plants export messenger RNA (mRNA) from the nucleus to the cytosol, where translation occurs (Fig. 1). Before export, mRNA undergoes essential processing steps, including intron splicing, the addition of a 5′ 7‐methylguanosine cap, and polyadenylation at the 3′ end. In the cytosol, the 5′ cap facilitates recruitment of translation initiation factors, enabling the preinitiation complex (PIC) to attach to the mRNA and scan for the start codon. The PIC scans in a strictly 5′‐to‐3′ direction until it reaches the start codon, usually the 5′‐most AUG codon, where the 80S ribosome assembles and translation initiates. The poly(A) tail ensures mRNA stability and interacts with the poly(A)‐binding protein, which associates with a translation initiation factor at the 5′ cap. This association circularizes the mRNA, which can increase translational efficiency, enhance tethering of initiation factors to the mRNA, and expedite ribosome recycling after translation termination (Jackson *et al*., 2010). Viral RNA without a 5′ cap or a poly(A) tail possesses alternative elements, such as internal ribosomal entry sites (IRESs), that stabilize the RNA and allow its efficient translation (Miras *et al*., 2017). In addition to expression of plant nuclear and viral genes, gene expression in plastids, where the nature of transcription and translation differs, is also of interest in biotechnology.

Controlling the amount of protein synthesized is crucial for efficient use of resources and response to environmental stimuli. This is, in part, regulated at the transcriptional level. However, regulation at the post‐transcriptional level is the faster and more direct response, which can then be sustained by transcriptional modulation. Post‐transcriptional regulation can occur through altered translational efficiency or mRNA stability. Regulatory elements on a specific RNA can exert control by themselves or through interaction with proteins or other RNAs, and this control can be exerted constitutively or in response to particular internal or external cues (Hershey *et al*., 2012).

In the case of mRNA, characterization of RNA species mostly focusses on the primary structure – the nucleotide sequence – which determines the amino acid sequence of the encoded polypeptide chain and its interaction with regulatory RNAs and the translation machinery. However, similarly to polypeptide chains, RNA forms higher order structures. It adopts secondary structures through intramolecular base pairing, tertiary structures that define its three‐dimensional conformation, and even quaternary structures involving the assembly of multiple interacting RNA molecules. RNA structure is also highly dynamic, constantly shifting between different conformations. The likelihood of adopting a particular structure is influenced by various factors, including proteins, other RNA molecules, small molecules, and external conditions, such as metal ion concentration, pH, crowding, and temperature. Both the nucleotide sequence, which ensures interaction specificity, and the multi‐tiered RNA structure with capacity to change are fundamental for the function of RNA regulatory elements, whether it is interaction with other molecules, sensing of environmental cues, catalysis, regulation of gene expression, RNA splicing, or RNA decay (Mustoe *et al*., 2014; Leamy *et al*., 2016; Ganser *et al*., 2019).

In this review, we explore RNA regulatory elements, highlighting their current biotechnological applications in plants and plant‐derived expression systems, while proposing potential avenues for their expanded use. RNA elements leading to replicon‐based systems, such as viral replicating expression systems, have been extensively discussed elsewhere (e.g. Peyret & Lomonossoff, 2015) and are beyond the scope of this review.

## II. Translational control as a tool for fine‐tuning gene expression

### 1. Start codon recognition

**Fig. 2:**
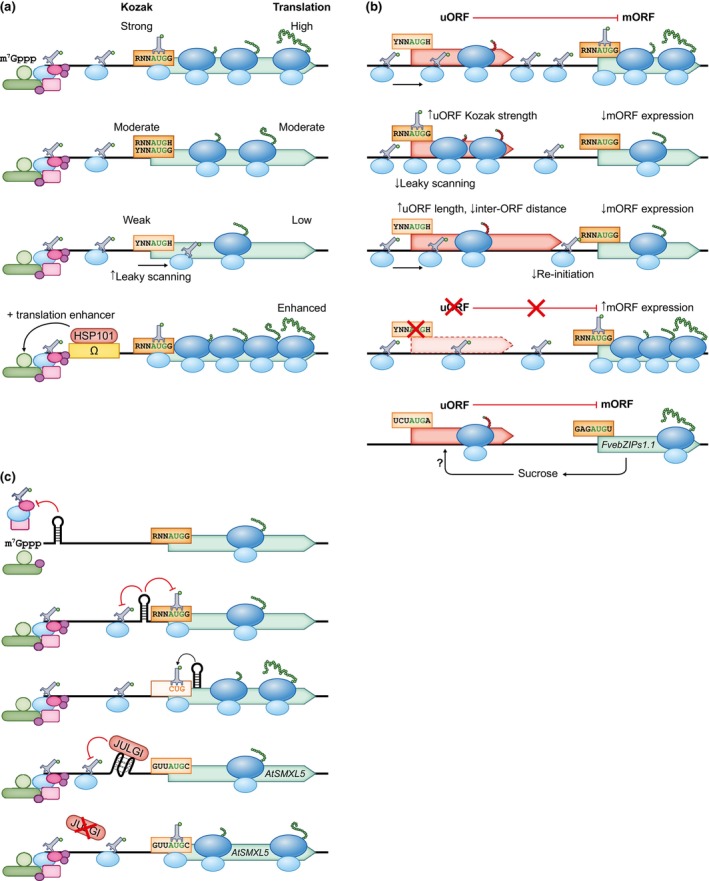
Fine‐tuning gene expression through translation initiation control. (a) Kozak sequence facilitates efficient translation, and it can vary in strength. Translation initiation can be further increased by translation enhancer sequences, such as the Tobacco mosaic virus Ω sequence, which interacts with HSP101. (b) Upstream open reading frames (uORFs) inhibit the translation of the main open reading frame (mORF). A stronger initiation context at the uORF, greater length, and shorter uORF–mORF distance increase its inhibitory effect. Some uORFs with highly conserved sequences, such as the *FvebZIPs1.1* uORF, inhibit mORF expression in response to a metabolite by an unknown mechanism. (c) Strong secondary structural elements in the 5′ untranslated region (UTR) can impede the recruitment of the 43S preinitiation complex (PIC) to the mRNA, its scanning, or translation initiation. A structure a short distance downstream of a noncanonical start codon can facilitate translation initiation. In the 5′ UTR of the *Arabidopsis thaliana SMXL5*, the RNA‐binding protein JULGI can induce an inhibitory G‐quadruplex. H, not guanine (A/C/U); R, purine (A/G); Y, pyrimidine (C/U). The initiation factors remain associated with the small ribosomal subunit until the recruitment of the 60S ribosomal subunit but are omitted from the illustration for simplicity. Blunt‐ended arrows indicate inhibition, and sharp arrows indicate enhancement or production.

Besides the start codon itself, efficient translation initiation is also facilitated by the sequences that flank the start codon, known as the Kozak sequence (Kozak, 1981, 1986, 1987a). Genetic, biochemical, and structural studies of yeast and animal PICs have revealed that recognition of the Kozak sequence involves initiation factors (eIF) 2, 1A, as well as the ribosomal protein uS7 and the 18S rRNA (Jackson *et al*., 2010; Brito Querido *et al*., 2024). Although the purine at position −3 and the guanine at +4 relative to the first nucleotide of the coding sequence (CDS) were found to have the strongest impact on start codon recognition in eukaryotes, nucleotides at positions −2 and −1 also have a great influence in plants (Kozak, 1987a; Lukaszewicz *et al*., 2000; Sugio *et al*., 2010). Poor context can allow bypass of the AUG by the scanning PIC and initiation at a downstream AUG (Fig. 2a), a phenomenon known as leaky scanning (Kozak, 1987a).

Design of protein‐producing expression systems as well as modification of endogenous genes should, hence, consider the translation initiation context. However, due to the divergence of the scanning complex, optimal Kozak sequences can vary between species: (GCCRCC**AUG**GC in monocots (and a similar sequence in nonflowering plants) and AAAAAA**AUG**GC in dicots (Joshi *et al*., 1997; Nakagawa *et al*., 2008; Rangan *et al*., 2008; Gupta *et al*., 2016)). Optimization of the Kozak sequence contributes towards high protein production. On the contrary, the choice of a suboptimal context, particularly at the positions −3 to −1 if the second codon has to be preserved, can be used to fine‐tune the efficiency of translational initiation, as demonstrated in mammalian cells (Xie *et al*., 2023). The strength of Kozak sequence variants has been assessed in dicot, monocot, and gymnosperm protoplasts and suspension cells (Lukaszewicz *et al*., 2000; Sugio *et al*., 2010). Adjustment of expression at the translational level may be desirable in situations where modification of the promoter region, to affect the transcript abundance, is undesirable or insufficient. Suboptimal contexts are also found around AUGs upstream of the main start codon; these initiate regulatory upstream open reading frames (uORFs).

### 2. Upstream open reading frames

In contrast to prokaryotes, eukaryotic transcripts tend to be monocistronic; nevertheless, a significant proportion of transcripts (over 30% in 11 species of plants; Von Arnim *et al*., 2014), have uORFs that precede the main ORFs (mORFs). The effect of uORFs on the mORF is generally inhibitory, as ribosomes are usually recycled after termination (Fig. 2b). However, small ribosomal subunits can remain associated with mRNA, resume scanning, and re‐initiate translation at a downstream ORF. The efficiency of re‐initiation is correlated with the following parameters: short distance between the uORF and the mORF (Kozak, 1987b); short uORF length (Luukkonen *et al*., 1995); and efficient elongation at the uORF (Kozak, 2001; Pöyry *et al*., 2004; Lin *et al*., 2019), which can be influenced by codon usage, strong secondary structure, or nature of the nascent peptide. It has therefore been hypothesized that efficient re‐initiation is conferred by undissociated initiation factors (probably eIF4G and eIF3) and acquisition of an initiator‐methionyl‐tRNA‐GTP‐eIF2 complex by the ribosome that has resumed scanning. Nevertheless, re‐initiation efficiency rarely exceeds 50% (Jackson *et al*., 2012). Translation of uORFs overlapping the mORFs is unlikely to allow re‐initiation at the mORF, although instances of backward scanning have been reported (Matsuda & Dreher, 2006; Wang *et al*., 2022; Dever *et al*., 2023). In addition, translation of uORFs can be bypassed through leaky scanning, which occurs more frequently if their start codon is non‐AUG or in a suboptimal context (Hinnebusch *et al*., 2016; Dever *et al*., 2023).

Notably, translation of uORFs can regulate mORF expression in response to internal conditions, and leveraging this mechanism has valuable potential in plant engineering. The transcription‐factor‐encoding *TBF1* is regulated by two conserved uORFs, which derepress the mORF translation in response to accumulation of uncharged tRNA^Phe^ during stress (Pajerowska‐Mukhtar *et al*., 2012). Certain metabolic genes are also regulated by uORFs that respond to the products of the associated metabolic pathway, for example, the synthesis of *S*‐adenosylmethionine decarboxylase in plants is negatively regulated by polyamines that act on the translation of two conserved uORFs. Translation of the first, very short uORF happens more frequently at low polyamine levels, bypasses the strongly inhibitory second uORF, and allows re‐initiation at the mORF (Hanfrey *et al*., 2005). Other examples of genes regulated by uORFs affected by small molecules are the sucrose‐regulated transcription factor gene *bZIP11* (Wiese *et al*., 2004; Xing *et al*., 2020), the phosphocholine‐regulated phosphocholine biosynthesis gene *XIPOTL1* (Tabuchi *et al*., 2006; Alatorre‐Cobos *et al*., 2012; Von Arnim *et al*., 2014) and the well‐known ascorbate‐responsive ascorbate biosynthesis gene *GGP* with a noncanonical ACG start codon (Laing *et al*., 2015).

For biotechnological purposes, translation can be further tuned by the introduction or modification of uORFs: those with a stronger Kozak sequence exert a stronger inhibitory effect on the mORF and vice versa (Xie *et al*., 2023). Manipulation of gene expression using uORFs is demonstrably feasible. Lettuce cultivars with an increased foliar ascorbic acid content and tolerance to oxidative stress were produced by disruption of the *GGP* uORF (Zhang *et al*., 2018). Strawberries with varying sugar contents were generated by mutating the sucrose‐responsive *FvebZIPs1.1* uORF and cross‐fertilizing the mutants with one another (Xing *et al*., 2020). Conversely, tunable inhibition of translation was achieved in rice by introduction of new uORFs or extension of existing uORFs (Xue *et al*., 2023).

### 3. Translation enhancer elements

In addition to Kozak sequence for optimal translation initiation, heterologous as well as endogenous gene expression can be increased by the introduction of translation enhancer elements, often derived from viruses. The most extensively used enhancer is the CA‐rich *Tobacco mosaic virus* Ω sequence, which binds heat shock protein 101 (HSP101) and increases the recruitment of eIF4F (Fig. 2a). This function overlaps with the function of 5′ cap and the poly(A) tail (Wells *et al*., 1998; Gallie, 2002). Another biotechnologically utilizable element comes from the *Alfalfa mosaic virus* RNA4 and showed a greater effect in rice (a monocot) than the Ω sequence (Jobling & Gehrke, 1987; Datla *et al*., 1993; Shen *et al*., 2023). Finally, synthetic enhancers have been developed, such as CA‐rich 5′ untranslated regions (UTRs) derived from random library screening (Kamura *et al*., 2005), synJ (Kanoria & Burma, 2012), ARC‐1, which is complementary to an internal 18S rRNA region (Akbergenov *et al*., 2004), and 5S0 (Peyret *et al*., 2019).

### 4. RNA structures as regulators of translation initiation

Much of our understanding of how RNA secondary structure affects eukaryotic translation initiation has been gained from studies in animal and yeast systems; these findings are relevant to plants as they share the general mechanism of translation initiation and should therefore be taken into account when designing expression systems or modifying genes. In yeast and mammals, the efficiency of translation initiation is normally reduced by the presence of strong secondary structure in particular regions of the 5′ UTR. For example, RNA stem–loop or G‐quadruplex structures located close to the 5′ end can inhibit the recruitment of the PIC to the mRNA. The helicase activity of eIF4A (and associated helicases) unwinds mRNA stem–loop structures as the PIC scans for the start codon (Brito Querido *et al*., 2024). Nevertheless, scanning can be obstructed by highly stable 5′‐UTR stem–loop or G‐quadruplex structures, which has been demonstrated in plant systems (Kozak, 1989; Kwok *et al*., 2015). Furthermore, stem–loop structures located just upstream of the start codon can reduce translation initiation efficiency. They may act as a physical barrier, obstructing the PIC, or alternatively, an RNA hairpin may displace the PIC from the start codon before GTP (guanosine triphosphate) hydrolysis by eIF2 and the subsequent joining of the 60S ribosomal subunit. Conversely, a stem–loop structure just downstream of a start site with a noncanonical start codon or poor context is known to enhance translational efficiency, likely by positioning or prolonging the positioning of the scanning PIC at the start site (Fig. 2c; Kozak, 1990; Wang *et al*., 2022; Cao *et al*., 2024).

Structural elements in the 5′ UTR can play a role in conditional translational regulation: for example, in vascular plants, the translation of *SMXL4/5*, which inhibits phloem differentiation, can be suppressed through the formation of 5′‐UTR G‐quadruplex, facilitated by the sucrose‐inducible RNA‐binding protein JULGI (Fig. 2c). Editing this system or adapting it to regulate other genes holds significant potential in improving crop yields (Cho *et al*., 2018; Cao *et al*., 2024).

A comparison of 5′ UTRs of highly expressed genes in plants has revealed that weak secondary structure of the 5′ UTR is linked to high‐expression levels (Peyret *et al*., 2019).

## III. Functional RNA domains

While the main role of mRNA is directing the synthesis of a polypeptide in a regulated manner, a number of mRNA species have additional sensory or catalytic functions that are instrumental in regulating their translation. A region of RNA can adopt a complex secondary and tertiary structure that forms a functional domain, analogous to a domain in proteins. An important fact to keep in mind when designing expression systems that rely on higher order structural elements is that the structure does not form in isolation and can be affected by other regions of the RNA as well as interacting proteins. A regulatory element may function effectively in its native context, but positioned next to a desired CDS or other regulatory elements in an artificial expression system, it may form an alternative structure, compromising its functionality. *In silico* predictions of local secondary structure may give indications of the compatibility of regulatory secondary structural elements with the designed expression system, but experimental verification ultimately provides the highest certainty.

### 1. Riboswitches

**Fig. 3:**
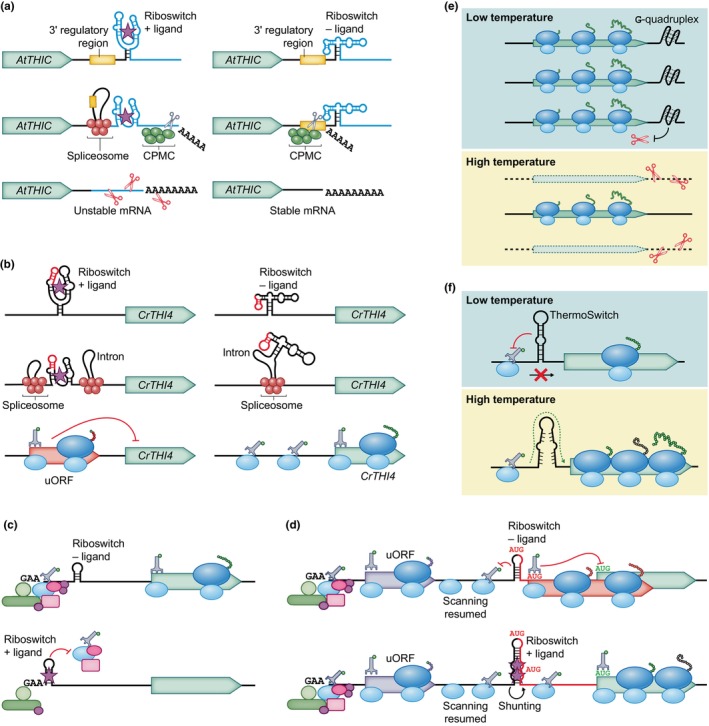
RNA switches responsive to ligands and temperature. (a) *Arabidopsis thaliana THIC* 3′ untranslated region (UTR) alternative splicing is regulated by a thiamine pyrophosphate (TPP) riboswitch. Binding of TPP leads to an alternative 3′ UTR (blue), removal of a 3′ regulatory region (yellow), and unstable mRNA. CPMC, cleavage and polyadenylation molecular complex. (b) *Chlamydomonas reinhardtii THI4* 5′ UTR alternative splicing is regulated by a TPP riboswitch. Binding of TPP leads to retention of an upstream open reading frame (uORF; pink) and inhibition of *THI4* translation. (c, d) Artificial riboswitches developed for expression systems based on wheat‐germ extract. Upon ligand binding, a riboswitch can inhibit translation by obstructing the recruitment of the 43S preinitiation complex (PIC: c). In a different construct, a riboswitch can activate translation by triggering ribosomal shunting (d). Following the translation of a uORF, the small ribosomal subunit restarts scanning. In the absence of a ligand, the scanning is hindered by an RNA hairpin or translation re‐initiates at one of two in‐frame decoy start codons, inhibiting the translation of the main ORF. In the presence of the ligand, the riboswitch forms a rigid stem, which allows scanning ribosomes to shunt over the structure, bypass decoy start codons, and initiate translation of the main ORF. The inhibitory uORF sequence with two in‐frame start codons is illustrated in pink. (e) G‐quadruplexes in the 3′ UTR stabilize the transcripts at low temperature. They do not form at higher temperatures, reducing the mRNA stability. (f) Regulation of translation by a ThermoSwitch. At low temperature, PIC scanning is obstructed. The ThermoSwitch adopts a relaxed conformation at a higher temperature, which is more easily unwound. The initiation factors remain associated with the small ribosomal subunit until the recruitment of the 60S ribosomal subunit but are omitted from the illustration for simplicity. Blunt‐ended arrows indicate inhibition.

RNA can fold into riboswitches: higher order structural elements that change conformation (‘switch’) in response to binding ions or small molecules. This alters the expression of the gene on the riboswitch‐controlled mRNA. In biotechnology, riboswitches confer inducibility of gene expression on the post‐transcriptional level, which is valuable in situations when constitutive expression is undesirable, such as when the protein of interest negatively impacts the expression system. In stably transformed plants, an inducible system allows protein production once the plant reaches a suitable size and avoids interference of the transgene expression with development or growth. While numerous classes of riboswitches have been described in bacteria, bacterial riboswitches are ineffective in eukaryotes (except in plastids) due to the inherent differences in translational mechanisms. Currently, the only known eukaryotic riboswitches bind the cofactor thiamine pyrophosphate (TPP) and its chemical analogues. They are often located within introns and regulate alternative splicing of mRNA, which can produce splice variants with premature stop codons, an alternative 3′ UTR that destabilizes the mRNA, or uORFs that inhibit translation of the mORF (Fig. 3a,b; Breaker, 2018; Croft *et al*., 2007).

TPP riboswitches have been used to control gene expression in *Arabidopsis* and tomato seedlings. However, their application in vascular plants appears limited so far, as ligand addition could reduce TPP‐riboswitch‐associated RNA abundance by up to fivefold (Wachter *et al*., 2007), while a significant reduction in *THIC* transcript levels was observed in a separate study only when thiamine auxotrophs were used (Bocobza *et al*., 2007). These findings suggest that the current biotechnological application of TPP riboswitches in vascular plants is mainly restricted to monitoring intracellular TPP levels (Bocobza & Aharoni, 2014).

On the contrary, TPP‐riboswitch‐based engineering has been more successful in the unicellular green alga *Chlamydomonas reinhardtii*. *Chlamydomonas* possesses two TPP‐responsive riboswitches, upstream of the genes *THIC* and *THI4*, which additionally also bind 4‐amino‐5‐hydroxymethyl‐2‐methylpyrimidine or 5‐hydroxyethyl‐4‐methylthiazole, respectively (Moulin *et al*., 2013). The *THI4* riboswitch controls alternative splicing that removes an inhibitory uORF in the absence of the ligand. In the engineered expression system, the *THI4* riboswitch is replaceable with alternative TPP riboswitches that have different ligand specificities and affinities, and it can successfully control the expression of heterologous genes (Mehrshahi *et al*., 2020).

Apart from one aptazyme (see the Catalytic RNAs section for further detail), no other riboswitches have been engineered in plants to control nuclear‐encoded genes. However, several artificially designed riboswitches have been assessed *in vitro* using wheat‐germ extract and are potentially applicable as *in vitro* biosensors. These *ab initio* riboswitches alter gene expression by mechanisms listed below and respond to a variety of ligands, such as theophylline, tetracycline, and riboflavin‐5′‐phosphate (Tabuchi & Yokobayashi, 2021):

- A strong RNA secondary structural element to obstruct ribosome association (Fig. 3c; Ogawa *et al*., 2018);
- Disruption of the proper folding of an IRES (Ogawa, 2011);
- Activation of ribosomal shunting (see Pooggin *et al*., 2000), allowing bypass of decoy start codons (Fig. 3d; Ogawa, 2013); and
- Inclusion of a 3′‐cap‐independent translational enhancer (CITE; see the Cap‐independent translation initiation section; Ogawa *et al*., 2017).

While artificial riboswitches in plants remain underexplored, many have been developed and tested in other eukaryotic systems, especially yeast and mammalian cells. In addition to alternative splicing similarly to native eukaryotic riboswitches (Culler *et al*., 2010), these artificial riboswitches utilize some noteworthy mechanisms, such as ligand‐induced −1 programmed ribosomal frameshifting (Lin & Chang, 2016) or a protein‐binding aptamer upstream of a non‐AUG start codon that stimulates translation (Horie *et al*., 2020). Future research may show whether these riboswitches are functional and sufficiently effective *in planta*.

### 2. Plant RNA structures conferring temperature‐sensitive regulation

While the RNA thermometers of bacteria and bacteriophages have been recognized since the 1980s (Altuvia *et al*., 1989), eukaryotic temperature‐sensitive RNA elements and their mechanism are less understood. Relatively recent studies have shown that plants possess RNA structures that confer responsiveness to temperature through effect on RNA stability or indeed translational efficiency (Chung *et al*., 2020; Thomas *et al*., 2022; Yang *et al*., 2022; Lastovka *et al*., 2024).

In plant mRNA, 3′ UTRs can respond to low temperatures by forming G‐quadruplexes that stabilize the transcript (Fig. 3e). This phenomenon was shown in *A. thaliana* seedlings subjected to a temperature of 4°C, along with its role in regulating root growth in cold temperatures. The RNA G‐quadruplexes regulated the mRNA abundance and had little effect on translational efficiency, independently of their location on the transcript. The effective stabilization of a reporter mRNA by this element reveals its capacity to regulate other mRNA species, whether endogenous or heterologous, and it therefore shows great promise for engineering of cold‐tolerant plants (Yang *et al*., 2022).

Control of plant translational efficiency by a temperature‐sensing RNA structure was also demonstrated in *A. thaliana*. A 5′‐UTR RNA hairpin, known as the ‘ThermoSwitch’, was identified in several transcripts that exhibited increased translational efficiency when the temperature rose by 10°C (17–27°C). This structure varied in sequence among different genes. Subsequent *in vitro* studies revealed that the shift range can be as narrow as 22–27°C to trigger enhanced protein synthesis of reporters under ThermoSwitch control (Chung *et al*., 2020). Eukaryotic ThermoSwitches differ from the bacterial RNA thermometers in multiple ways, in part due to the different nature of prokaryotic and eukaryotic translational initiation. Bacterial RNA thermometers change conformation or gradually melt at warmer temperatures and thus reveal (or obscure) the ribosome‐binding site. On the contrary, plant ThermoSwitches have a seemingly dual mechanistic function: below the threshold temperature, they impede the scanning of the PIC, which travels from the 5′ end of the mRNA, from accessing the start codon; above the threshold temperature, the ThermoSwitch adopts a relaxed conformation, which enables the PIC to reach the start codon (Fig. 3f). The partially molten hairpin additionally enhances translation, possibly by temporarily impeding the incoming PIC while the previous complex initiates translation (Chung *et al*., 2020; Thomas *et al*., 2022). The molecular mechanism is the subject of ongoing research. A recent study demonstrated that the *PIF7* ThermoSwitch, when integrated into an *Agrobacterium*‐mediated transient expression system, enables temperature‐sensitive regulation of gene expression in *Nicotiana benthamiana*, indicating its potential as a biotechnological tool for homogeneous induction of gene expression, independent of chemical inducers or suppressors (Lastovka *et al*., 2024).

### 3. Catalytic RNAs

Analogously to enzymes, RNA can form domains – ribozymes – that catalyse chemical reactions. While the ribozyme active site consists of RNA, some ribozymes, such as ribosomes, contain protein components (Cech, 2000). The catalytic functions of most naturally occurring ribozymes are RNA cleavage or splicing *in cis*. Self‐splicing introns found in mitochondria and plastids rely on *cis*‐acting ribozymes that splice the pre‐mRNA sequence independently of spliceosomes (Mukhopadhyay & Hausner, 2021). *Cis*‐cleaving ribozymes are used by viruses, satellite viruses, and viroids, including the plant pathogens *Avocado sunblotch viroid* and *Tobacco ringspot virus* satellite RNA to cleave their RNA during replication (De la Peña *et al*., 2017). Ribozyme motifs are widespread in genomes of all kingdoms of life, and their identification in plant genomes has led to the discovery of retrozymes, nonautonomous retrotransposons that encode circular RNA (Cervera *et al*., 2016; De la Peña *et al*., 2017). Of the 10 families of *cis*‐cleaving ribozymes, the hammerhead ribozyme is the most extensively characterized (De la Peña *et al*., 2017).

**Fig. 4:**
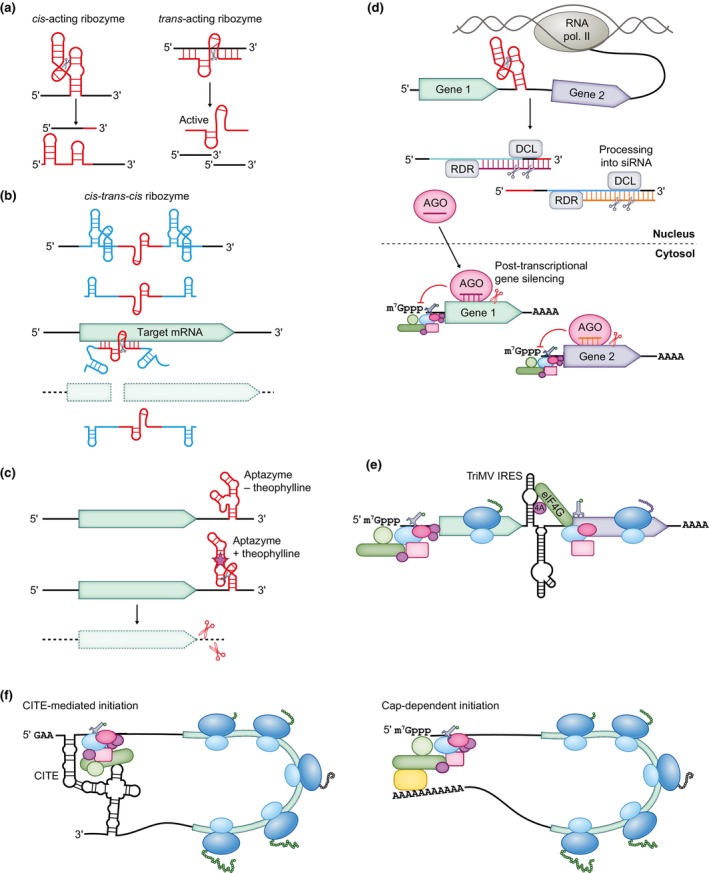
Catalytic RNA and cap‐independent translation initiation. (a) Ribozymes acting *in cis* and *in trans*. A hammerhead ribozyme structure is illustrated. (b) A *cis‐trans‐cis* ribozyme transcribed from a suppression cassette. The *trans*‐acting ribozyme is excised from the construct and targets the gene of interest for cleavage. (c) An aptazyme cleaves plant mRNA *in cis* in response to theophylline. The cleaved mRNA is then degraded. (d) A suppression construct that contains two coding sequences separated by a ribozyme. The cleaved transcript is processed into small interfering RNA (siRNA) using an RNA‐dependent RNA polymerase (RDR) and a Dicer‐like endoribonuclease (DCL). The generated siRNA associates with Argonaute (AGO) and inhibits the translation or cleaves the mRNA of the target genes. (e) The *Triticum mosaic virus* (TriMV) internal ribosomal entry site (IRES) can bring about internal translation initiation, thereby allowing the translation of both coding sequences in an artificial bicistronic construct. The function is independent of initiation factor 4E, but requires initiation factor 4G (eIF4G), which interacts with the upper stem of the first IRES hairpin, and initiation factor 4A. (f) Cap‐independent translational enhancer (CITE) facilitates translation of uncapped mRNA *in vitro*. Initiation factors, and possibly also the 43S preinitiation complex (PIC), are recruited to the 3′ CITE and delivered to the 5′ end, from where the PIC scans. Pairing of the 5′ and 3′ structures circularizes the mRNA, similarly to the circularization of capped and polyadenylated mRNA (below). Blunt‐ended arrows indicate inhibition.

Naturally occurring ribozymes can be converted to *trans*‐acting ribozymes by separating the catalytic region from the substrate region and modifying the substrate‐recognition sequences to target a specific RNA species (Fig. 4a). *Trans*‐acting ribozymes are true catalysts, as they retain their activity following cleavage of the substrate. A minimal hammerhead ribozyme sequence active *in vitro* has been identified, which consists of the catalytic core flanked by substrate‐recognition sequences that direct cleavage 3′ of NHH (N is any nucleotide and H is any nucleotide apart from guanine; Peng *et al*., 2021). However, the cleavage activity of the minimal ribozyme is reduced *in vivo* at low concentrations of magnesium. The activity in these conditions is improved by the addition of tertiary stabilizing motifs, which bring about interaction between the ribozyme loops and increase the effectiveness both *in vitro* and *in vivo* (Carbonell *et al*., 2011). Further refinements include, for example, the construction of *cis*‐*trans*‐*cis* ribozyme cassettes, where self‐cleaving ribozymes liberate a *trans*‐acting ribozyme without flanking sequences that could interfere with folding or substrate specificity (Fig. 4b; Bussière *et al*., 2003).

*Trans*‐acting ribozymes have been directed against viral or viroidal RNA to generate resistant transgenic plants and successfully reduced the severity of symptoms. Targeting the negative‐sense RNA of positive‐strand RNA viruses and viroids conferred stronger protection (Atkins *et al*., 1995; Yang *et al*., 1997; Huttner *et al*., 2001). Although the use of a minimal ribozyme showed success against viruses and viroids, the effectiveness varied between plant species and the inheritance of the resistance phenotype was irregular due to RNA silencing interfering with ribozyme accumulation (Yang *et al*., 1997; Han *et al*., 2000). Multimeric ribozymes, ribozymes fused with satellite RNA that exploit the viral replication machinery, and ribozymes with tertiary stabilizing motifs were more successful than minimal ribozymes (Kwon *et al*., 1997; Carbonell *et al*., 2011).

Reduction of endogenous gene expression using *trans*‐acting ribozymes had varying levels of success. An approximately fourfold reduction in target mRNA abundance was achieved in transgenic maize with a multimeric ribozyme (Merlo *et al*., 1998). A *cis*‐*trans*‐*cis* stabilized ribozyme cassette in transgenic potato attained a reduction of targeted mRNA abundance to 36–50% in a third of the transgenic plants (Bussière *et al*., 2003). While *trans*‐acting ribozymes offer precision in cleavage and lower risk of off‐target effects, RNA silencing has a stronger inhibitory effect (Akashi *et al*., 2005).

Synthetic ligand‐dependent ribozymes – aptazymes – have been engineered by coupling an aptamer domain with a ribozyme domain. Similarly to riboswitches, binding of a ligand to the aptamer leads to a structural rearrangement of the RNA, which activates or inactivates the ribozyme domain. A *cis* aptazyme is usually placed in the 3′ UTR so that translation initiation is not inhibited by the higher order structures, and the conditional cleavage destabilizes the mRNA (Win & Smolke, 2007; Peng *et al*., 2021). Aptazymes responsive to multiple signals can be integrated into logic gates (Win & Smolke, 2008). *Trans*‐acting hammerhead aptazymes have been developed as well (Zhou *et al*., 2023). While aptazymes have chiefly been implemented in bacteria, yeast, and mammalian cells, a theophylline‐sensing hammerhead aptazyme successfully controlled the expression of plant nuclear‐encoded genes (Fig. 4c; Shanidze *et al*., 2020). This offers a prospect of developing new inducible expression systems, for example for plant molecular farming, which may be informed by the aptazymes developed for use in other organisms. Alternative aptamers would be necessary for applications in theophylline‐producing plant species. In addition to controlling gene expression, aptazymes on reporter genes can serve as noninvasive sensors of intracellular protein, metabolite concentration, or temperature (Win & Smolke, 2007; Park *et al*., 2019). In yeast, a theophylline aptazyme on *GFP* (green fluorescent protein gene) was used to monitor the intracellular xanthine concentration (Win & Smolke, 2007), and it is conceivable that aptamers sensing alternative ligands could be applied for this purpose in plants.

Self‐cleaving ribozymes have been combined with RNA silencing (see the RNA silencing section) to reduce plant gene expression: truncation of sense and antisense transcripts to retain them in the nucleus induced RNA silencing (Fig. 4d; Buhr *et al*., 2002). Considering this application, it could be interesting to fuse an aptazyme sequence with a target plant gene sequence, even an artificial *trans*‐acting small interfering RNA gene, to induce RNA silencing that is not reliant on conditionally active promoters, similarly to a system constructed in mammalian cells by fusion of an aptazyme with a primary microRNA sequence (Kumar *et al*., 2009). In another application combining two mechanisms, ribozymes were used in the production of guide RNAs (gRNAs) for CRISPR‐Cas9 plant genome editing: They allow the placement of multiple gRNA sequences on a single, potentially conditionally regulated transcript, which is cleaved by *cis*‐acting ribozymes into the individual parts (He *et al*., 2017).

### 4. Cap‐independent translation initiation

RNA viruses that replicate in the cytoplasm are excluded from nuclear capping machinery. Viruses that do not encode their own capping enzymes produce uncapped RNAs that initiate translation via cap‐independent mechanisms. Moreover, some RNA viruses that possess viral capping machinery still produce subgenomic RNAs that remain uncapped and are translated through alternative initiation strategies (Kneller *et al*., 2006). Functional RNA structures can enable translation initiation independently of the 5′ cap. An IRES is an RNA structure that serves as a platform for ribosome recruitment and assembly at an adjacent start codon. This recruitment avoids the canonical scanning of the PIC for start codons from the 5′ end. IRESs have been found in plant viruses, such as *Triticum mosaic virus*, the IRES of which is over 700 nt long. It forms two bulged stem–loop structures in the 5′ leader and has features similar to picornaviral IRESs (Roberts *et al*., 2015, 2017; Miras *et al*., 2017; Jaramillo‐Mesa *et al*., 2019, 2022). Even some cellular genes, such as the maize heat shock gene *HSP101* or the tobacco *HSF1*, contain an IRES, which ensures that their translation is sustained when cap‐dependent translation is downregulated (Dorokhov *et al*., 2002; Son & Park, 2023). An IRES can direct the translation of an additional ORF on the same RNA, a phenomenon that is well characterized in animal viruses (e.g. *Dicistroviridae*; Martinez‐Salas *et al*., 2018). However, the mechanisms of IRES‐dependent translation and stress‐induced inhibition of cap‐dependent translation in plants are not yet well understood, and the mechanistic nature of plant virus intergenic IRESs, such as the one present in crucifer‐infecting *Tobamovirus* (crTMV), which is short and has a simple secondary structure, has been called into question (Dorokhov *et al*., 2002; Miras *et al*., 2017; Son & Park, 2023).

While virus‐derived IRESs have been successfully deployed in animal cells and yeast to produce bicistronic transcripts, there has been mixed success in plants (Wang & Marchisio, 2021). IRES sequences derived from both animal and plant viruses led to co‐expression of genes from bicistronic transcripts *in vitro*, in transfected protoplasts, as well as in transgenic plants (Urwin *et al*., 2000; Dorokhov *et al*., 2002). The *Triticum mosaic virus* IRES facilitated bicistronic reporter gene translation in wheat‐germ extract independently of the 5′ cap, the cap‐binding eIF4E, or the poly(A) tail (Fig. 4e; Roberts *et al*., 2015, 2017). Although a crTMV IRES could direct bicistronic gene co‐expression in transgenic rice, a StopGo sequence (see StopGo sequence) was markedly more effective (Ha *et al*., 2010). This IRES was also successfully used to coproduce coronaviral proteins in *Agrobacterium*‐mediated transient expression experiments (Moon *et al*., 2022). However, in attempts to construct bicistronic expression vectors for *Chlamydomonas*, IRESs derived from an array of plant and animal viruses were found to be ineffective (Onishi & Pringle, 2016). The IRES from *Encephalomyocarditis virus* could not bring about bicistronic expression in transgenic rice (Jung *et al*., 2011).

Other elements used by RNA viruses to avoid the requirement for a 5′ cap or a poly(A) tail are CITEs, which include the *Barley yellow dwarf virus* translation element or a variety of CITEs in *Tombusviridae* (Meulewaeter *et al*., 1998; Guo *et al*., 2001; Fabian & White, 2004; Wang *et al*., 2009; Zuo *et al*., 2010; Simon & Miller, 2013; Truniger *et al*., 2017). These structurally diverse elements bind translation initiation factors, particularly the cap‐interacting eIF4G or eIF4E, and some interact directly with ribosomal subunits. The effect of most 3′ CITEs depends on a long‐distance base‐pairing with a 5′‐end structure on the same RNA to deliver the recruited translation machinery to the 5′ end (Fig. 4f). In contrast to the IRES mechanism, the ribosomes recruited by a CITE scan for a start codon from the 5′ end rather than entering internally (Rakotondrafara *et al*., 2006; Nicholson *et al*., 2010). The fact that CITEs are functional when placed in the genomes of other viruses demonstrates their modularity and presents a prospect to use them in artificial constructs (Nicholson *et al*., 2010, 2013; Miras *et al*., 2014).

CITEs enhance the existing capacity of the translational machinery to initiate at uncapped mRNA, which is particularly useful in *in vitro* translation systems: CITEs have been used to achieve high translational efficiency of uncapped mRNA with short UTRs in wheat‐germ extract (Ogawa *et al*., 2014). CITEs increase the translation of uncapped mRNA delivered to protoplasts, although capping the mRNA led to higher expression levels (Guo *et al*., 2001; Chattopadhyay *et al*., 2011; Fan *et al*., 2012; Gao *et al*., 2012). On the contrary, in *Agrobacterium*‐mediated transient transformation experiments, inclusion of synthetic CITEs did not increase protein yield compared with CITE‐free constructs, possibly due to unsuitable sequence context or because the element did not further enhance the translation of 5′‐capped mRNA (Peyret *et al*., 2019). It would be interesting to find out if CITEs or IRESs reinforce gene expression under stress conditions, and if so, if this capacity could be exploited in engineering stress‐tolerant crops.

## IV. Other RNA elements

### 1. StopGo sequence

**Fig. 5:**
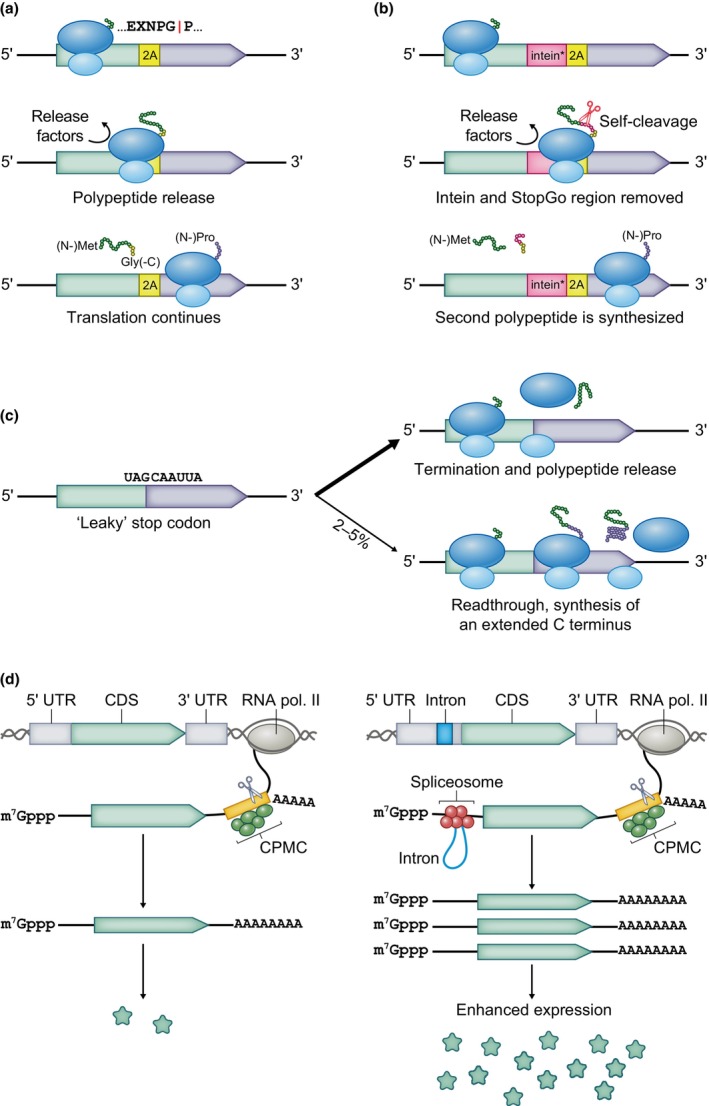
StopGo element, stop codon readthrough, and intron‐mediated enhancement. (a) StopGo element, such as the *Foot‐and‐mouth disease virus* 2A, allows coproduction of two polypeptide chains encoded in the same open reading frame. As the StopGo element is translated, the ribosome releases the polypeptide and resumes translation. The C terminus of the released polypeptide contains the 2A sequence. (b) Encoding a modified intein domain (intein*) upstream of the StopGo element allows the synthesized polypeptide to self‐cleave, removing the intein‐ and 2A‐derived C terminus. (c) ‘Leaky’ stop codons allow a small proportion of ribosomes to read through and continue synthesizing a protein isoform with an extended C terminus. (d) Enhancing introns can augment expression by multiple mechanisms that increase transcript accumulation and translation efficiency. CPMC, cleavage and polyadenylation molecular complex.

In addition to IRESs, an RNA element that ensures simultaneous synthesis of two or more proteins encoded on a single transcript is the StopGo sequence, first identified in the *Foot‐and‐mouth disease virus*, where it is known as the 2A sequence. Originally believed to be an autocatalytic cleavage site in the viral polyprotein (Ryan *et al*., 1991), the StopGo element encodes a polypeptide sequence that causes a skip in the formation of a glycine‐proline peptide bond (‘ribosomal stutter’), release of the nascent chain by release factors, and continuation of translation, which generates a second polypeptide with an N‐terminal proline (Fig. 5a; Atkins *et al*., 2007; Donnelly *et al*., 2001; Doronina *et al*., 2008; Sharma *et al*., 2012). A 30‐aa‐encoding StopGo sequence is the shortest length that produces efficient stutter independently of the flanking sequences; reducing its length can tune the ratio of joined and separated products (Minskaia *et al*., 2013). The major appeal of this element is its capacity to achieve co‐ordinated protein production, which is difficult to achieve otherwise, even when the genes have identical promoters (Halpin *et al*., 1999). StopGo sequences are effective when introduced to a variety of eukaryotic cells or *in vitro* translation systems, including plant‐derived ones (Halpin *et al*., 1999; Donnelly *et al*., 2001; Ha *et al*., 2010; Burén *et al*., 2012; Zhang *et al*., 2017; Lee *et al*., 2020; Larsen *et al*., 2023), which makes them very useful biotechnological tools. The disadvantage is that the StopGo‐derived C terminus is linked to the upstream‐encoded protein, which can disrupt its folding, interactions, localization, or activity. This problem is alleviated by encoding a modified intein domain upstream of the StopGo sequence that cleaves off the StopGo‐derived C terminus (Fig. 5b; Zhang *et al*., 2017).

### 2. Stop codon readthrough

Specific stop codons can be redefined to encode an amino acid instead of termination, which allows a proportion of ribosomes to translate beyond the ‘leaky’ stop codon, yielding protein variants with alternative C termini (Fig. 5c). These events occur in multiple virus families, and hundreds of potential sites exist in plant transcriptomes (Firth *et al*., 2011; Sahoo *et al*., 2022; Zhang *et al*., 2024). Readthrough is facilitated *in cis* by the stop codon context or an adjacent secondary structure, but the stimulatory mechanisms are not clearly understood (Firth *et al*., 2011; Palma & Lejeune, 2021). The more efficient use of nucleotide sequence allows viral genome compression, while in biotechnology, sequence length constraints are a lot more relaxed. Yet, stop codon readthrough has found multiple biotechnological applications, where a readthrough rate of 2–5% was induced by a **UAG**CAAUUA stop codon context. It was used to produce *Tobamovirus* particles with heterologous polypeptides on the surface, which have potential pharmaceutical use or confer heavy metal resistance (Hamamoto *et al*., 1993; Fujiyama *et al*., 2006; Shingu *et al*., 2006). A reporter system was developed in *Chlamydomonas* where the CDSs of the investigated gene and a fluorescence gene were fused at a leaky stop codon, producing both a functional protein and a nonfunctional but trackable fluorescent fusion protein (Caspari, 2020).

### 3. Expression‐enhancing introns

Once transcribed, pre‐mRNA undergoes further processing to become mature mRNA before being exported out of the nucleus. These processing steps include the splicing of introns by the spliceosome, a ribonucleoprotein complex. In addition to alternative splicing, which produces mRNA with different combinations of exons and can affect expression by adding or removing regulatory RNA elements, the very presence of an intron can increase expression on the transcriptional and post‐transcriptional levels (Fig. 5d). Introns of certain plant genes were shown to exert effects specific to tissue, developmental stage, or stress. The phenomenon is called intron‐mediated enhancement (IME) and has been used in biotechnology of plants ranging from algae to angiosperms, even though its precise mechanisms, of which there are multiple, so far remain unclear. The proposed mechanisms include recruitment of transcription factors, chromatin remodelling, reduction in RdRP‐dependent RNA silencing, and inhibition of premature RNA cleavage and polyadenylation. So far, the clearest mechanism is association with proteinaceous *trans*‐factors, including exon junction complexes and serine‐arginine‐rich proteins, deposited during intron splicing, which can enhance nucleus export and translational efficiency (Pydiura & Blume, 2023; Zhong *et al*., 2023).

An expression‐enhancing intron has to be located in the proximity of the transcription start site and possess efficient splice sites, the nature of which can differ among plant clades (Zhong *et al*., 2023). On the contrary, 3′‐UTR introns are known to stimulate mRNA degradation by nonsense‐mediated decay, a pathway that removes mRNA with premature stop codons (Kertesz *et al*., 2006). The strength of IME varies with a range of intronic properties, such as length, position, and orientation, as well as intronic and flanking sequence composition, including the presence of certain motifs (Chung *et al*., 2006; Pydiura & Blume, 2023). Clarification of the molecular mechanisms and how they relate to specific intron properties will aid in the design of effectively enhanced or conditionally expressed constructs.

### 4. 3′ UTR elements

Most genes that are efficiently expressed require 3′ end polyadenylation of the mRNA, which promotes both its transcription and translation, ensures mRNA stability and export from the nucleus, and protects the transcript from RdRP‐mediated RNA silencing in plants. Cleavage and polyadenylation are signalled by *cis*‐acting elements, which are located chiefly downstream of the CDS and influence the gene expression level. In both stably integrated and transiently expressed plant expression constructs, the cleavage and polyadenylation signals are present in ‘terminators’ (distinct from prokaryotic terminators), also known as 3′ regulatory regions, the use of which in plant biotechnology was reviewed by Bernardes & Menossi (2020).

The cleavage and polyadenylation site determines the 3′ UTR of the transcript, which can contain a range of elements with stabilizing, destabilizing, or translation‐modulating effect. The elements can form higher order structures or constitute binding sites for silencing RNAs or proteins, most of which are adaptors for effector proteins or mediate co‐translational protein–protein interaction. Study of the protein‐binding sites, the RNA‐binding proteins, and the sequence properties can guide the engineering of 3′ UTRs, but it is complicated by the markedly different behaviour of the 3′ UTR elements in isolation and in a wider sequence context (Mayr, 2017). Identification of 3′ UTR inhibitory elements has enabled their deletion from the genome, which increased the expression of the modified endogenous genes (Wang *et al*., 2024). Also important for 3′ UTR design is the finding that, despite the stabilization of mRNA by G‐quadruplex structures, the strength of plant 3′ UTR secondary structure is inversely correlated with mRNA stability (Yang *et al*., 2022; Zhang *et al*., 2024). Another intriguing finding was the suppression of translation caused by a 3′‐UTR G‐quadruplex structure in the *A. thaliana* gene *HIRD11* (Yang *et al*., 2020; Cao *et al*., 2024).

The 3′ UTRs in gene expression constructs can contain viral elements, such as the previously discussed CITEs, to increase the protein yield. A noteworthy element is the Y‐shaped structure in the *Cowpea mosaic virus* RNA‐2. This element enhances mRNA accumulation likely by protecting the mRNA from 3′‐exonuclease‐mediated degradation and has been effectively utilized in high‐expression transient expression systems (Meshcheriakova *et al*., 2014; Peyret *et al*., 2019).

### 5. RNA silencing

RNA silencing, the suppression of expression directed in a sequence‐specific manner by *trans*‐acting regulatory RNAs, is an important means of gene expression regulation involved in a wide range of processes from development, to stress responses, to antiviral defence. The process is set in motion by double‐stranded RNA (dsRNA), which is processed into fragments, associates with a multiprotein complex, and brings about cleavage or translational inhibition of complementary RNA. RNA silencing constitutes a useful alternative to gene deletion in cases where a deletion compromises plant growth or reproduction, and it has been extensively used to study gene function and to improve crop or decorative plants. RNA silencing in biotechnology was reviewed in detail by Ossowski *et al*. (2008); Tiwari *et al*. (2014); Verma & Modgil (2024).

### 6. RNA modification

RNA base modifications, such as to N^6^‐methyladenosine, can change the stability and translation of mRNA. The exerted effect on gene expression is important for a variety of processes, including stem cell fate determination, fruit ripening, and stress responses. Therefore, manipulation of RNA modification holds great promise in crop improvement, as reviewed by Tang *et al*. (2023) and Tang & Wang (2024).

## V. RNA elements in engineered chloroplasts

An alternative to editing the nuclear genome is the modification of the chromosome in plastids. Benefits of this strategy, such as high‐expression levels (in part due to the high plastome copy number per cell) and the absence of mechanisms for silencing heterologous genes in plastids, may for certain purposes, outweigh its greater technical challenges (Bock, 2015). The wide variety of metabolic pathways occurring in plastids presents the option to boost the biosynthesis of useful endogenous metabolites, such as carotenoids, or introduce enzymes that use plastidial metabolic substrates to synthesize foreign metabolic products, such as bioplastics (Wurbs *et al*., 2007; Bohmert‐Tatarev *et al*., 2011; Bock, 2015). Heterologous genes can also be expressed in other types of plastids, particularly amyloplasts and chromoplasts, to produce proteins of interest in nongreen tissues, such as roots and fruit, but the overall gene expression is markedly lower (Valkov *et al*., 2011; Zhang *et al*., 2012; Caroca *et al*., 2013; Bock, 2015).

### 1. Start codon recognition

**Fig. 6:**
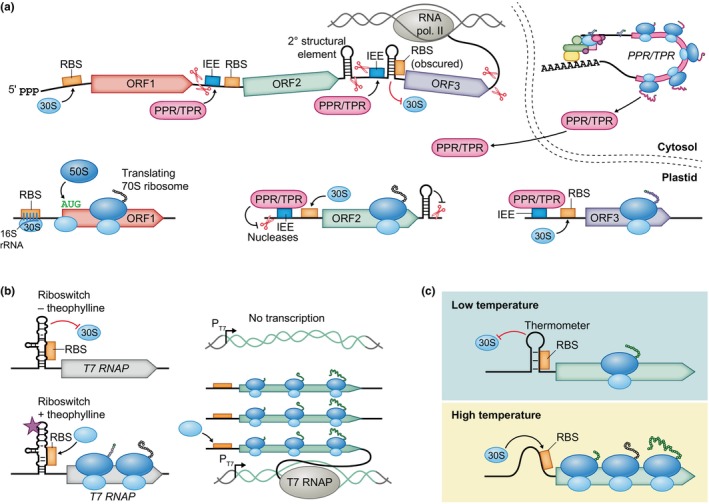
Gene expression in plastids. (a) In plastids, multiple genes can be cotranscribed into a polycistronic mRNA, which is processed by nucleases (red scissors). 30S ribosomal subunits are recruited to ribosome‐binding sites (RBS), often by binding the Shine–Dalgarno sequence with their 16S rRNA, and the 50S subunit associates once the start codon is reached. Intercistronic expression elements (IEE) are the binding sites for pentatricopeptide/tetratricopeptide repeat (PPR/TPR) proteins, which are encoded in the nucleus and imported into the plastid. Binding of PPRs/TPRs increases mRNA stability and remodels its secondary structure, which may reveal ribosome‐binding sites or nuclease sites. mRNA is additionally protected from degradation by secondary structural elements. ORF, open reading frame. RNA pol., RNA polymerase. (b) In an artificial theophylline‐responsive expression system, a riboswitch controls plastidial gene expression by suppressing/enabling the synthesis of T7 RNA polymerase (T7 RNAP). The gene of interest is under the control of a T7 promoter (P_T7_) and transcribed only by T7 RNAP. (c) An RNA thermometer controls gene expression by obscuring the RBS at low temperatures and revealing it at higher temperatures. Black blunt‐ended arrows show inhibition that aids gene expression, and red blunt‐ended arrows indicate inhibition that obstructs gene expression.

Plastids are believed to have arisen from ancient Cyanobacteria (Martin & Kowallik, 1999); as these are prokaryotes, their translational regulation differs substantially from the one in the eukaryotic cytosol. In prokaryotes, the small ribosomal subunit interacts with a ribosome‐binding site a short distance upstream of the CDS (Fig. 6a). An efficient ribosome‐binding site often contains the purine‐rich Shine–Dalgarno (SD) sequence, which forms base pairs with the 3′ end of the 16S rRNA (the anti‐SD sequence), and has a weak secondary structure (Wen *et al*., 2021; Bryant *et al*., 2023). While the SD interaction with the anti‐SD in the 16S rRNA is typically important for efficient translation in plastids, Cyanobacteria and chloroplasts contain a large proportion of genes that lack an SD sequence (Scharff *et al*., 2011; Lomsadze *et al*., 2018). While their 16S rRNA contains the conserved prokaryotic anti‐SD sequence, the 3′ end tends to form a secondary structure that obscures it (Weiner *et al*., 2019). Successfully expressed heterologous genes inserted into plastid genomes are generally designed to include an SD sequence. Importantly for biotechnology, introduction of a modified 16S rRNA gene with an accessible anti‐SD sequence can enhance the translation of a plastidial gene of interest that has an SD sequence (Weiner *et al*., 2019). Moreover, bacteriophage T7 gene 10 5′ UTR is used in plastids to enhance translation of the downstream CDS of interest (Olins *et al*., 1988; Bock, 2015).

### 2. Polycistronic transcripts and mRNA processing

Another distinctive feature of prokaryotic and plastidial gene expression is the cotranscription of multiple genes onto a single polycistronic mRNA (Fig. 6a). This is particularly beneficial for engineering of metabolic pathways or producing protein complexes, as multiple genes can be joined into an operon for cotranscription (Bohmert‐Tatarev *et al*., 2011; Lu *et al*., 2013; Lin *et al*., 2014; Fuentes *et al*., 2016; Long *et al*., 2018). In contrast to bacteria, plastids often process mRNA: removing introns and cleaving polycistronic transcripts with nucleases into monocistronic fragments (Barkan, 2011; Bock, 2022, 2015). The intercistronic nuclease cleavage is evidently not site‐specific, and monocistronic fragments probably are stabilized degradation intermediates (Barkan, 2011). The expression of some genes relies on or is improved by the nucleolytic processing into monocistronic fragments. This is partly because the unprocessed mRNA contains inhibitory secondary structures that hinder expression (Barkan *et al*., 1994; Hirose & Sugiura, 1997; Felder *et al*., 2001). Other genes, even heterologous operons, are expressed even without the accumulation of monocistronic fragments (Staub & Maliga, 1995; Barkan, 2011, 1988; Yu *et al*., 2020). Because the dependence of translational efficiency on nucleolytic processing is difficult to predict, constructed operons usually contain intercistronic expression elements (IEEs) between genes to increase the reliability of expression (Zhou *et al*., 2007; Bock, 2022). These elements can recruit nuclear‐encoded RNA‐binding pentatricopeptide/tetratricopeptide repeat (PPR/TPR) proteins, which protect the bound mRNA fragment from exonucleases, and remodel the local secondary structure, thus derepressing translation and possibly promoting intercistronic cleavage by exposing endonuclease sites (Felder *et al*., 2001; Pfalz *et al*., 2009; Prikryl *et al*., 2011; Hammani *et al*., 2012; Legen *et al*., 2018; Macedo‐Osorio *et al*., 2021). It is important to consider compatibility of IEE with native PPR/TPR proteins when introducing a heterologous sequence (Bock, 2022). Additionally, it is also important to account for the fact that an excessive presence of IEEs can sequester PPR/TPR proteins, potentially destabilizing endogenous transcripts (Legen *et al*., 2018). This issue can be addressed by using alternative IEEs in the inserted or modified sequences and encoding a PPR/TPR protein in the nuclear genome that specifically recognizes these alternative IEEs (Rojas *et al*., 2019; Yu *et al*., 2019, 2020).

To achieve high protein synthesis levels, the engineered plastidial expression systems incorporate 3′ UTR sequences downstream of CDSs. These sequences protect the transcript or processed transcript fragment by forming a strong mRNA stem–loop structure (Stern & Gruissem, 1987; Zhou *et al*., 2007). An IEE also protects the 3′ end even of fragments lacking a stabilizing secondary structure (Legen *et al*., 2018). The 3′ UTRs used in such systems can be derived from existing plastidial genes, such as *rbcL* (RuBisCO large chain gene), and may originate from other species (Lu *et al*., 2013).

### 3. Conditional expression

Conditional expression of plastidial genes may be desirable in scenarios where high levels of their expression could be harmful to the plant, when gene expression is needed in specific tissues, or when suppressing the expression of a gene essential for survival or development is necessary. Conditional regulation can be achieved using RNA elements for post‐transcriptional control. For example, a modified PPR‐protein‐binding site that recruits an artificial PPR protein encoded in the nucleus under the control of an inducible or tissue‐specific promoter (Rojas *et al*., 2019; Yu *et al*., 2019). This method has demonstrated success in conditionally stimulating the expression of plastidial genes, with a 20‐fold increase in reporter accumulation upon induction and a 15‐fold reduction in nontarget tissues. In a similar system in *Chlamydomonas*, the *THI4* riboswitch was used to control the production of a nuclear‐encoded TPR protein (Ramundo & Rochaix, 2015).

The introduction of a synthetic theophylline‐responsive riboswitch achieved tightly regulated inducible expression without the need to modify the nuclear genome, but with *c*. 10 times lower maximal expression level than a riboswitch‐free construct (Verhounig *et al*., 2010). The expression was enhanced in an improved system utilizing the bacteriophage T7 RNA polymerase promoter, which is not recognized by the plastidial RNA polymerases. The T7 promoter controlled the target gene, and its transcription was activated by induction of a riboswitch‐controlled T7 RNA polymerase gene in the plastidial chromosome (Fig. 6b; Agrawal *et al*., 2022; Emadpour *et al*., 2015). Despite this improvement, the system achieved a narrower expression range, with only sevenfold difference between induced and baseline expression, compared with the broader range achieved using the modified PPR protein system. Computational tools for design of prokaryotic riboswitches built from diverse RNA aptamers could guide the development of plastid‐based phytosensors (Espah Borujeni *et al*., 2016), but their design will be complicated by the differences in riboswitch effectiveness between bacteria and chloroplasts, as was the case with glycine‐, adenine‐, and thiamine‐pyrophosphate‐responsive riboswitches (Verhounig *et al*., 2010).

The *Chlamydomonas* chloroplast contains an RNA thermometer that obscures the SD sequence of *psaA* at 25°C and derepresses translation at higher temperatures. The thermometer was successfully used to control heterologous protein synthesis, and an engineered extension of the stem suppressed expression at the lower temperature while enabling inducibility at 35–40°C (Fig. 6c; Chung *et al*., 2023). As with riboswitches, a collection of thermometer elements that were functional in *E. coli* did not confer temperature‐sensitive expression in chloroplasts, although a possible reason is the lack of 5′ UTR stabilization rather than the ineffectiveness of the thermometers.

In bacteria, small noncoding RNAs (sRNAs) regulate gene expression by forming duplexes with mRNA, which can inhibit the mRNA translation or target it for degradation (Ahmed *et al*., 2018). A variety of sRNA species have been detected in chloroplasts, some antisense to chloroplastidial genes, but their effect on gene expression requires further investigation (Anand & Pandi, 2021). Exploring the inducible production of sRNA complementary to the ribosome‐binding site of target genes could provide a potential strategy for conditionally suppressing gene expression in plastids.

It is noteworthy that despite the prokaryotic origin of plastids, their gene expression mechanisms have marked differences from the ones in bacteria, and this ought to be considered when adapting bacterial RNA elements for use in plastids.

## VI. Perspectives

The discovery and study of the variety of RNA regulatory elements have opened up new avenues for their application as powerful tools in plant biotechnology, with benefits spanning from basic research to agricultural innovation. Primarily, but not exclusively, acting at the post‐transcriptional level, these elements enable modulation of heterologous and endogenous gene expression both *in cis* and *in trans*, facilitate co‐ordinated gene expression, and enable conditional regulation. Plant molecular genetics research, particularly of differential gene expression and RNA structure, is poised to expand this repertoire further, perhaps with the aid of artificial intelligence to predict RNA structure and interaction with proteins. Beyond plant genomes, plant viruses and subviral pathogens serve as a rich resource of functional RNA elements, optimized through evolution to manipulate host machinery and drive efficient gene expression. Plant viruses have additionally been adapted into autonomously replicating vectors, which we have not discussed in this review (Peyret & Lomonossoff, 2015).

Among the diversity of identified RNA elements (Table 1), many have untapped potential in plant biotechnology. First, the feasibility of employing certain elements, such as the newly characterized thermosensitive RNA elements or aptazymes, in plant biotechnology has already been demonstrated (Shanidze *et al*., 2020; Lastovka *et al*., 2024). Further optimization will enhance their practicality for real‐world application. Second, some RNA elements that have been successfully tested in animal cells or yeast are yet to be explored in plants. These include aptazyme‐based logic gates and a broader range of aptamers for translation‐regulating riboswitches which could expand the toolkit for gene regulation in plant systems (Win & Smolke, 2008).

**Table 1:** Biotechnologically useful RNA regulatory elements.

Element	Location	Effect	References
**Translational control**			
Kozak sequence	Translation initiation site	Crucial for translational efficiency. Tunable. Efficient sequences vary among plant clades	Lukaszewicz *et al*. (2000); Sugio *et al*. (2010)
uORF	5′ UTR	Inhibitory, but tunable. Its translational efficiency inversely correlates with the main ORF expression	Von Arnim *et al*. (2014); Zhang *et al*. (2018); Xing *et al*. (2020); Xue *et al*. (2023)
Translational enhancer	5′ UTR	Increased translational efficiency	Gallie (2002); Akbergenov *et al*. (2004); Kamura *et al*. (2005); Kanoria & Burma (2012); Peyret *et al*. (2019); Shen *et al*. (2023)
Secondary structural elements	5′ UTR, translation initiation site, 3′ UTR	A strong 5′‐UTR structure inhibits translation. The effect can be conditional. Possibly enhanced translation initiation at weak sites by hairpins just downstream	Wang *et al*. (2022); Cao *et al*. (2024)
**Functional RNA domains**			
Riboswitch	Splice sites *in vivo*, UTRs *in vitro*	Inducible/suppressible gene expression by a variety of ligands	Mehrshahi *et al*. (2020); Tabuchi & Yokobayashi (2021)
Temperature‐sensitive elements	UTRs	Inducible gene expression by temperature	Thomas *et al*. (2022); Yang *et al*. (2022); Lastovka *et al*. (2024)
Ribozyme	3′ UTR or *trans*‐acting	RNA decay, potentially amplified by RNA silencing	Peng *et al*. (2021)
Aptazyme	3′ UTR	RNA decay inducible by a ligand	Shanidze *et al*. (2020); Peng *et al*. (2021)
Internal ribosomal entry site	5′ UTR, intercistronic region	Expression of genes from polycistronic transcripts	Urwin *et al*. (2000); Dorokhov *et al*. (2002); Roberts *et al*. (2015); Moon *et al*. (2022)
Cap‐independent translational enhancer	5′ and 3′ UTR	Efficient translation of uncapped RNA *in vitro*	Ogawa *et al*. (2014)
**Other RNA elements**			
StopGo sequence	Coding sequence	Coproduction of multiple proteins encoded on the same transcript	Halpin (2005); Zhang *et al*. (2017)
Stop codon readthrough	Translation termination site	Coproduction of protein variants with alternative C termini	Hamamoto *et al*. (1993); Fujiyama *et al*. (2006); Shingu *et al*. (2006); Caspari (2020)
Expression‐enhancing intron	Proximity to transcription initiation site	Increased transcript accumulation and possibly also translational efficiency	Pydiura & Blume (2023); Zhong *et al*. (2023)
3′ regulatory region	3′ end	Essential for transcript stability, nuclear export, and translation	Bernardes & Menossi (2020)
CPMV RNA‐2 Y‐loop	3′ UTR	Enhanced transient gene expression, probably by mRNA stabilization.	Meshcheriakova *et al*. (2014); Peyret *et al*. (2019)
miRNA and ta‐siRNA	*Trans*‐acting	RNA decay, translational inhibition, transcriptional inhibition	Ossowski *et al*. (2008); Tiwari *et al*. (2014); Verma & Modgil (2024)
RNA base modification	UTRs or CDS	Altered RNA stability or translational efficiency, alternative polyadenylation	Tang *et al*. (2023); Tang & Wang (2024)
**RNA elements in plastids**			
Shine–Dalgarno sequence	Translation initiation site in plastidial mRNA	Efficient translation initiation at a specific site	Bock (2015, 2022); Weiner *et al*. (2019)
T7 gene 10 5′ UTR	5′ UTR in plastidial mRNA	Increased translational efficiency	Olins *et al*. (1988); Bock (2015)
Intercistronic expression elements	Intergenic regions in plastidial operons	Transcript stabilization and gene co‐expression. Can rely on inducible proteins	Bock (2015, 2022)
Riboswitches and thermometers	5′ UTR in plastidial mRNA	Inducible gene expression by ligands or temperature	Verhounig *et al*. (2010); Chung *et al*. (2023)

CDS, coding sequence; miRNA, microRNA; mRNA, messenger RNA; ta‐siRNA, *trans*‐acting small interfering RNA; UTR, untranslated region.

Furthermore, some RNA elements require further investigation but show strong potential as valuable biotechnological tools. RNA localization elements or ‘postcodes’, primarily characterized in cereal endosperm cells, interact with *trans*‐acting factors that guide them to a specific subcellular location. This targeting is mainly achieved through interaction with cytoskeletal motor proteins (Tian *et al*., 2020). In addition to temporal control of expression conferred by inducible elements, RNA postcodes could offer means of intracellular spatial control and improve the assembly of multiprotein complexes or import of the encoded proteins into organelles. Additionally, long‐distance mobility has been described of mRNA and other major RNA classes through phloem, even to distinct organs. RNA elements, such as tRNA‐like structures or polypyrimidine motifs, have been linked to long‐distance transport; however, the underlying mechanism remains unclear and requires further investigation (Kehr & Kragler, 2018). It has been suggested that mobile RNA could be used in phytosensors to transmit a signal from the sensing tissue to readily accessible parts of the plant (Mahmudul *et al*., 2022). In addition, 3′‐end tRNA‐like structures have also been linked to RNA stabilization and translational enhancement, likely in a conditional manner, and further insight into their function and mechanism will allow us to evaluate their biotechnological potential (Wu *et al*., 2022).

Adaptation of RNA elements for biotechnological purposes is not always straightforward: elements like viral IRESs are functional in their native setting, but less effective in artificial systems (see the Cap‐independent translation initiation section) and would benefit from optimization. Sequence context, plant tissue, and the plant species can influence the effectiveness of certain RNA elements, which has to be taken into account before their introduction into the expression system of interest. While gaps exist in our knowledge of many RNA elements as well as of plant molecular genetics and stress responses, filling them will pave way for the development of improved expression systems and their application in plant engineering.

## Competing interests

None declared.

## Disclaimer

The New Phytologist Foundation remains neutral with regard to jurisdictional claims in maps and in any institutional affiliations.

## References

[nph70400-bib-0001] Agrawal S , Karcher D , Ruf S , Erban A , Hertle AP , Kopka J , Bock R . 2022. Riboswitch‐mediated inducible expression of an astaxanthin biosynthetic operon in plastids. Plant Physiology 188: 637–652. 34623449 10.1093/plphys/kiab428 PMC8774745

[nph70400-bib-0002] Ahmed W , Hafeez MA , Mahmood S . 2018. Identification and functional characterization of bacterial small non‐coding RNAs and their target: A review. Gene Reports 10: 167–176.

[nph70400-bib-0003] Akashi H , Matsumoto S , Taira K . 2005. Gene discovery by ribozyme and siRNA libraries. Nature Reviews Molecular Cell Biology 6: 413–422. 15956980 10.1038/nrm1646

[nph70400-bib-0004] Akbergenov RZ , Zhanybekova SS , Kryldakov R , Zhigailov A , Polimbetova N , Hohn T , Iskakov B . 2004. ARC‐1, a sequence element complementary to an internal 18S rRNA segment, enhances translation efficiency in plants when present in the leader or intercistronic region of mRNAs. Nucleic Acids Research 32: 239–247. 14718549 10.1093/nar/gkh176 PMC373286

[nph70400-bib-0005] Alatorre‐Cobos F , Cruz‐Ramírez A , Hayden CA , Pérez‐Torres C‐A , Chauvin A‐L , Ibarra‐Laclette E , Alva‐Cortés E , Jorgensen RA , Herrera‐Estrella L . 2012. Translational regulation of arabidopsis XIPOTL1 is modulated by phosphocholine levels via the phylogenetically conserved upstream open reading frame 30. Journal of Experimental Botany 63: 5203–5221. 22791820 10.1093/jxb/ers180

[nph70400-bib-0006] Altuvia S , Kornitzer D , Teff D , Oppenheim AB . 1989. Alternative mRNA structures of the cIII gene of bacteriophage determine the rate of its translation initiation. Journal of Molecular Biology 210: 265–280. 2532257 10.1016/0022-2836(89)90329-x

[nph70400-bib-0007] Anand A , Pandi G . 2021. Noncoding RNA: an insight into chloroplast and mitochondrial gene expressions. Lifestyles 11: 12548. 10.3390/life11010049 PMC7828403 33450961

[nph70400-bib-0008] Atkins D , Young M , Uzzell S , Kelly L , Fillatti J , Gerlach WL . 1995. The expression of antisense and ribozyme genes targeting citrus exocortis viroid in transgenic plants. Journal of General Virology 76: 1781–1790. 9049383 10.1099/0022-1317-76-7-1781

[nph70400-bib-0009] Atkins JF , Wills NM , Loughran G , Wu C‐Y , Parsawar K , Ryan MD , Wang C‐H , Nelson CC . 2007. A case for ‘StopGo’: reprogramming translation to augment codon meaning of GGN by promoting unconventional termination (stop) after addition of glycine and then allowing continued translation (go). RNA 13: 803–810. 17456564 10.1261/rna.487907 PMC1869043

[nph70400-bib-0010] Barkan A . 1988. Proteins encoded by a complex chloroplast transcription unit are each translated from both monocistronic and polycistronic mRNAs. EMBO Journal 7: 2637–2644. 2460341 10.1002/j.1460-2075.1988.tb03116.x PMC457051

[nph70400-bib-0011] Barkan A . 2011. Expression of plastid genes: organelle‐specific elaborations on a prokaryotic scaffold. Plant Physiology 155: 1520–1532. 21346173 10.1104/pp.110.171231 PMC3091090

[nph70400-bib-0012] Barkan A , Walker M , Nolasco M , Johnson D . 1994. A nuclear mutation in maize blocks the processing and translation of several chloroplast mRNAs and provides evidence for the differential translation of alternative mRNA forms. EMBO Journal 13: 3170–3181. 8039510 10.1002/j.1460-2075.1994.tb06616.x PMC395209

[nph70400-bib-0013] Bernardes WS , Menossi M . 2020. Plant 3' regulatory regions from mRNA‐encoding genes and their uses to modulate expression. Frontiers in Plant Science 11: 1252. 32922424 10.3389/fpls.2020.01252 PMC7457121

[nph70400-bib-0014] Bock R . 2015. Engineering plastid genomes: methods, tools, and applications in basic research and biotechnology. Annual Review of Plant Biology 66: 211–241. 10.1146/annurev-arplant-050213-040212 25494465

[nph70400-bib-0015] Bock R . 2022. Transplastomic approaches for metabolic engineering. Current Opinion in Plant Biology 66: 102185. 35183927 10.1016/j.pbi.2022.102185

[nph70400-bib-0016] Bocobza S , Adato A , Mandel T , Shapira M , Nudler E , Aharoni A . 2007. Riboswitch‐dependent gene regulation and its evolution in the plant kingdom. Genes & Development 21: 2874–2879. 18006684 10.1101/gad.443907 PMC2049190

[nph70400-bib-0017] Bocobza SE , Aharoni A . 2014. Small molecules that interact with RNA: riboswitch‐based gene control and its involvement in metabolic regulation in plants and algae. The Plant Journal 79: 693–703. 24773387 10.1111/tpj.12540

[nph70400-bib-0018] Bohmert‐Tatarev K , McAvoy S , Daughtry S , Peoples OP , Snell KD . 2011. High levels of bioplastic are produced in fertile transplastomic tobacco plants engineered with a synthetic operon for the production of polyhydroxybutyrate. Plant Physiology 155: 1690–1708. 21325565 10.1104/pp.110.169581 PMC3091132

[nph70400-bib-0019] Breaker RR . 2018. Riboswitches and translation control. Cold Spring Harbor Perspectives in Biology 10: a032797. 29844057 10.1101/cshperspect.a032797 PMC6211393

[nph70400-bib-0020] Brito Querido J , Díaz‐López I , Ramakrishnan V . 2024. The molecular basis of translation initiation and its regulation in eukaryotes. Nature Reviews Molecular Cell Biology 25: 168–186. 38052923 10.1038/s41580-023-00624-9

[nph70400-bib-0207] Browning KS . 2014. Cytoplasm: Translational Apparatus. In: Molecular Biology. New York, NY: Springer, 1–19.

[nph70400-bib-0021] Browning KS , Webster C , Roberts J , Ravel J . 1992. Identification of an isozyme form of protein synthesis initiation factor 4F in plants. Journal of Biological Chemistry 267: 10096–10100. 1577779

[nph70400-bib-0022] Bryant OJ , Lastovka F , Powell J , Chung BY‐W . 2023. The distinct translational landscapes of Gram‐negative Salmonella and Gram‐positive Listeria. Nature Communications 14: 8167. 10.1038/s41467-023-43759-1 PMC10710512 38071303

[nph70400-bib-0023] Buhr T , Sato S , Ebrahim F , Xing A , Zhou Y , Mathiesen M , Schweiger B , Kinney A , Staswick P , Clemente T . 2002. Ribozyme termination of RNA transcripts down‐regulate seed fatty acid genes in transgenic soybean. The Plant Journal 30: 155–163. 12000452 10.1046/j.1365-313x.2002.01283.x

[nph70400-bib-0024] Burén S , Ortega‐Villasante C , Ötvös K , Samuelsson G , Bakó L , Villarejo A . 2012. Use of the foot‐and‐mouth disease virus 2A peptide co‐expression system to study intracellular protein trafficking in Arabidopsis. PLoS ONE 7: e51973. 23251667 10.1371/journal.pone.0051973 PMC3522588

[nph70400-bib-0025] Bussière F , Ledû S , Girard M , Héroux M , Perreault J‐P , Matton DP . 2003. Development of an efficient *cis*‐*trans*‐cis ribozyme cassette to inactivate plant genes. Plant Biotechnology Journal 1: 423–435. 17134401 10.1046/j.1467-7652.2003.00039.x

[nph70400-bib-0026] Cao X , Zhang Y , Ding Y , Wan Y . 2024. Identification of RNA structures and their roles in RNA functions. Nature Reviews Molecular Cell Biology 25: 784–818. 38926530 10.1038/s41580-024-00748-6

[nph70400-bib-0027] Carbonell A , Flores R , Gago S . 2011. *Trans*‐cleaving hammerhead ribozymes with tertiary stabilizing motifs: *in vitro* and *in vivo* activity against a structured viroid RNA. Nucleic Acids Research 39: 2432–2444. 21097888 10.1093/nar/gkq1051 PMC3064770

[nph70400-bib-0028] Caroca R , Howell KA , Hasse C , Ruf S , Bock R . 2013. Design of chimeric expression elements that confer high‐level gene activity in chromoplasts. The Plant Journal 73: 368–379. 23004223 10.1111/tpj.12031

[nph70400-bib-0029] Caspari OD . 2020. Introduction of a leaky stop codon as molecular tool in *chlamydomonas reinhardtii* . PLoS ONE 15: e0237405. 32817702 10.1371/journal.pone.0237405 PMC7440625

[nph70400-bib-0030] Cech TR . 2000. The ribosome is a ribozyme. Science 289: 878–879. 10960319 10.1126/science.289.5481.878

[nph70400-bib-0031] Cervera A , Urbina D , de la Peña M . 2016. Retrozymes are a unique family of non‐autonomous retrotransposons with hammerhead ribozymes that propagate in plants through circular RNAs. Genome Biology 17: 1–16. 27339130 10.1186/s13059-016-1002-4 PMC4918200

[nph70400-bib-0032] Chattopadhyay M , Shi K , Yuan X , Simon AE . 2011. Long‐distance kissing loop interactions between a 3′ proximal y‐shaped structure and apical loops of 5′ hairpins enhance translation of saguaro cactus virus. Virology 417: 113–125. 21664637 10.1016/j.virol.2011.05.007 PMC3152624

[nph70400-bib-0033] Cho H , Cho HS , Nam H , Jo H , Yoon J , Park C , Dang TVT , Kim E , Jeong J , Park S *et al*. 2018. Translational control of phloem development by RNA g‐quadruplex–JULGI determines plant sink strength. Nature Plants 4: 376–390. 29808026 10.1038/s41477-018-0157-2

[nph70400-bib-0034] Chung BY , Balcerowicz M , Di Antonio M , Jaeger KE , Geng F , Franaszek K , Marriott P , Brierley I , Firth AE , Wigge PA . 2020. An RNA thermoswitch regulates daytime growth in arabidopsis. Nature Plants 6: 522–532. 32284544 10.1038/s41477-020-0633-3 PMC7231574

[nph70400-bib-0035] Chung BY , Simons C , Firth AE , Brown CM , Hellens RP . 2006. Effect of 5′UTR introns on gene expression in *Arabidopsis thaliana* . BMC Genomics 7: 1–13. 16712733 10.1186/1471-2164-7-120 PMC1482700

[nph70400-bib-0036] Chung KP , Loiacono FV , Neupert J , Wu M , Bock R . 2023. An RNA thermometer in the chloroplast genome of chlamydomonas facilitates temperature‐controlled gene expression. Nucleic Acids Research 51: 11386–11400. 37855670 10.1093/nar/gkad816 PMC10639063

[nph70400-bib-0037] Croft MT , Moulin M , Webb ME , Smith AG . 2007. Thiamine biosynthesis in algae is regulated by riboswitches. Proceedings of the National Academy of Sciences, USA 104: 20770–20775. 10.1073/pnas.0705786105 PMC2410077 18093957

[nph70400-bib-0038] Culler SJ , Hoff KG , Smolke CD . 2010. Reprogramming cellular behavior with RNA controllers responsive to endogenous proteins. Science 330: 1251–1255. 21109673 10.1126/science.1192128 PMC3171693

[nph70400-bib-0039] Datla RS , Bekkaoui F , Hammerlindl JK , Pilate G , Dunstan DI , Crosby WL . 1993. Improved high‐level constitutive foreign gene expression in plants using an AMV RNA4 untranslated leader sequence. Plant Science 94: 139–149.

[nph70400-bib-0040] De la Peña M , García‐Robles I , Cervera A . 2017. The hammerhead ribozyme: a long history for a short RNA. Molecules 22: 78. 28054987 10.3390/molecules22010078 PMC6155905

[nph70400-bib-0041] Dever TE , Ivanov IP , Hinnebusch AG . 2023. Translational regulation by uORFs and start codon selection stringency. Genes & Development 37: 474–489. 37433636 10.1101/gad.350752.123 PMC10393191

[nph70400-bib-0042] Donnelly ML , Luke G , Mehrotra A , Li X , Hughes LE , Gani D , Ryan MD . 2001. Analysis of the aphthovirus 2A/2B polyprotein ‘cleavage’ mechanism indicates not a proteolytic reaction, but a novel translational effect: a putative ribosomal ‘skip’. Journal of General Virology 82: 1013–1025. 11297676 10.1099/0022-1317-82-5-1013

[nph70400-bib-0043] Dorokhov YL , Skulachev MV , Ivanov PA , Zvereva SD , Tjulkina LG , Merits A , Gleba YY , Hohn T , Atabekov JG . 2002. Polypurine (a)‐rich sequences promote cross‐kingdom conservation of internal ribosome entry. Proceedings of the National Academy of Sciences, USA 99: 5301–5306. 10.1073/pnas.082107599 PMC122764 11959981

[nph70400-bib-0044] Doronina VA , Wu C , de Felipe P , Sachs MS , Ryan MD , Brown JD . 2008. Site‐specific release of nascent chains from ribosomes at a sense codon. Molecular and Cellular Biology 28: 4227–4239. 18458056 10.1128/MCB.00421-08 PMC2447138

[nph70400-bib-0045] Emadpour M , Karcher D , Bock R . 2015. Boosting riboswitch efficiency by RNA amplification. Nucleic Acids Research 43: e66. 25824954 10.1093/nar/gkv165 PMC4446413

[nph70400-bib-0046] Espah Borujeni A , Mishler DM , Wang J , Huso W , Salis HM . 2016. Automated physics‐based design of synthetic riboswitches from diverse RNA Aptamers. Nucleic Acids Research 44: 1–13. 26621913 10.1093/nar/gkv1289 PMC4705656

[nph70400-bib-0047] Fabian MR , White KA . 2004. 5′–3′ RNA‐RNA interaction facilitates cap‐and poly (a) tail‐independent translation of tomato bushy stunt virus mRNA: a potential common mechanism for tombusviridae. Journal of Biological Chemistry 279: 28862–28872. 15123633 10.1074/jbc.M401272200

[nph70400-bib-0048] Fan Q , Treder K , Miller WA . 2012. Untranslated regions of diverse plant viral RNAs vary greatly in translation enhancement efficiency. BMC Biotechnology 12: 1–10. 22559081 10.1186/1472-6750-12-22 PMC3416697

[nph70400-bib-0049] Felder S , Meierhoff K , Sane AP , Meurer J , Driemel C , Plücken H , Klaff P , Stein B , Bechtold N , Westhoff P . 2001. The nucleus‐encoded HCF107 gene of Arabidopsis provides a link between intercistronic RNA processing and the accumulation of translation‐competent psbH transcripts in chloroplasts. Plant Cell 13: 2127–2141. 11549768 10.1105/TPC.010090 PMC139456

[nph70400-bib-0050] Firth AE , Wills NM , Gesteland RF , Atkins JF . 2011. Stimulation of stop codon readthrough: frequent presence of an extended 3′ RNA structural element. Nucleic Acids Research 39: 6679–6691. 21525127 10.1093/nar/gkr224 PMC3159437

[nph70400-bib-0051] Fuentes P , Zhou F , Erban A , Karcher D , Kopka J , Bock R . 2016. A new synthetic biology approach allows transfer of an entire metabolic pathway from a medicinal plant to a biomass crop. eLife 5: e13664. 27296645 10.7554/eLife.13664 PMC4907697

[nph70400-bib-0052] Fujiyama K , Saejung W , Yanagihara I , Nakado J , Misaki R , Honda T , Watanabe Y , Seki T . 2006. In planta production of immunogenic poliovirus peptide using tobacco mosaic virus‐based vector system. Journal of Bioscience and Bioengineering 101: 398–402. 16781468 10.1263/jbb.101.398

[nph70400-bib-0053] Gallie DR . 2002. The 5′‐leader of tobacco mosaic virus promotes translation through enhanced recruitment of eIF4F. Nucleic Acids Research 30: 3401–3411. 12140325 10.1093/nar/gkf457 PMC137081

[nph70400-bib-0054] Ganser LR , Kelly ML , Herschlag D , Al‐Hashimi HM . 2019. The roles of structural dynamics in the cellular functions of RNAs. Nature Reviews Molecular Cell Biology 20: 474–489. 31182864 10.1038/s41580-019-0136-0 PMC7656661

[nph70400-bib-0055] Gao F , Kasprzak W , Stupina VA , Shapiro BA , Simon AE . 2012. A ribosome‐binding, 3′ translational enhancer has a t‐shaped structure and engages in a long‐distance RNA‐RNA interaction. Journal of Virology 86: 9828–9842. 22761367 10.1128/JVI.00677-12 PMC3446580

[nph70400-bib-0056] Guo L , Allen EM , Miller WA . 2001. Base‐pairing between untranslated regions facilitates translation of uncapped, nonpolyadenylated viral RNA. Molecular Cell 7: 1103–1109. 11389856 10.1016/s1097-2765(01)00252-0

[nph70400-bib-0057] Gupta P , Rangan L , Ramesh TV , Gupta M . 2016. Comparative analysis of contextual bias around the translation initiation sites in plant genomes. Journal of Theoretical Biology 404: 303–311. 27316311 10.1016/j.jtbi.2016.06.015

[nph70400-bib-0058] Ha S‐H , Liang YS , Jung H , Ahn M‐J , Suh S‐C , Kweon S‐J , Kim D‐H , Kim Y‐M , Kim J‐K . 2010. Application of two bicistronic systems involving 2A and IRES sequences to the biosynthesis of carotenoids in rice endosperm. Plant Biotechnology Journal 8: 928–938. 20649940 10.1111/j.1467-7652.2010.00543.x

[nph70400-bib-0059] Halpin C . 2005. Gene stacking in transgenic plants–the challenge for 21^st^ century plant biotechnology. Plant Biotechnology Journal 3: 141–155. 17173615 10.1111/j.1467-7652.2004.00113.x

[nph70400-bib-0060] Halpin C , Cooke SE , Barakate A , Amrani AE , Ryan MD . 1999. Self‐processing 2A‐polyproteins–a system for co‐ordinate expression of multiple proteins in transgenic plants. The Plant Journal 17: 453–459. 10205902 10.1046/j.1365-313x.1999.00394.x

[nph70400-bib-0061] Hamamoto H , Sugiyama Y , Nakagawa N , Hashida E , Matsunaga Y , Takemoto S , Watanabe Y , Okada Y . 1993. A new tobacco mosaic virus vector and its use for the systemic production of angiotensin‐i‐converting enzyme inhibitor in transgenic tobacco and tomato. Bio/Technology 11: 930–932. 7763916 10.1038/nbt0893-930

[nph70400-bib-0062] Hammani K , Cook WB , Barkan A . 2012. RNA binding and RNA remodeling activities of the half‐a‐tetratricopeptide (HAT) protein HCF107 underlie its effects on gene expression. Proceedings of the National Academy of Sciences, USA 109: 5651–5656. 10.1073/pnas.1200318109 PMC3326456 22451905

[nph70400-bib-0063] Han S , Wu Z , Yang H , Wang R , Yie Y , Xie L , Tien P . 2000. Ribozyme‐mediated resistance to rice dwarf virus and the transgene silencing in the progeny of transgenic rice plants. Transgenic Research 9: 195–203. 11032368 10.1023/a:1008904230223

[nph70400-bib-0064] Hanfrey C , Elliott KA , Franceschetti M , Mayer MJ , Illingworth C , Michael AJ . 2005. A dual upstream open reading frame‐based autoregulatory circuit controlling polyamine‐responsive translation. Journal of Biological Chemistry 280: 39229–39237. 16176926 10.1074/jbc.M509340200

[nph70400-bib-0065] He Y , Zhang T , Yang N , Xu M , Yan L , Wang L , Wang R , Zhao Y . 2017. Self‐cleaving ribozymes enable the production of guide RNAs from unlimited choices of promoters for CRISPR/Cas9 mediated genome editing. Journal of Genetics and Genomics 44: 469–472. 28958488 10.1016/j.jgg.2017.08.003 PMC5736383

[nph70400-bib-0066] Hershey JW , Sonenberg N , Mathews MB . 2012. Principles of translational control: an overview. Cold Spring Harbor Perspectives in Biology 4: a011528. 23209153 10.1101/cshperspect.a011528 PMC3504442

[nph70400-bib-0067] Hinnebusch AG , Ivanov IP , Sonenberg N . 2016. Translational control by 5′‐untranslated regions of eukaryotic mRNAs. Science 352: 1413–1416. 27313038 10.1126/science.aad9868 PMC7422601

[nph70400-bib-0068] Hirose T , Sugiura M . 1997. Both RNA editing and RNA cleavage are required for translation of tobacco chloroplast ndhD mRNA: a possible regulatory mechanism for the expression of a chloroplast operon consisting of functionally unrelated genes. EMBO Journal 16: 6804–6811. 9362494 10.1093/emboj/16.22.6804 PMC1170284

[nph70400-bib-0069] Horie F , Endo K , Ito K . 2020. Artificial protein‐responsive riboswitches upregulate non‐AUG translation initiation in yeast. ACS Synthetic Biology 9: 1623–1631. 32531157 10.1021/acssynbio.0c00206

[nph70400-bib-0070] Huttner E , Tucker W , Vermeulen A , Ignart F , Sawyer B , Birch R . 2001. Ribozyme genes protecting transgenic melon plants against potyviruses. Current Issues in Molecular Biology 3: 27–34. 11471972

[nph70400-bib-0071] Jackson RJ , Hellen CU , Pestova TV . 2010. The mechanism of eukaryotic translation initiation and principles of its regulation. Nature Reviews Molecular Cell Biology 11: 113–127. 20094052 10.1038/nrm2838 PMC4461372

[nph70400-bib-0072] Jackson RJ , Hellen CU , Pestova TV . 2012. Termination and post‐termination events in eukaryotic translation. In: Advances in protein chemistry and structural biology, vol. 86. Cambridge, MA, USA: Academic Press, 45–93. 10.1016/B978-0-12-386497-0.00002-5 22243581

[nph70400-bib-0073] Jaramillo‐Mesa H , Fischer E , Rakotondrafara AM . 2022. Multiple *cis*‐acting polypyrimidine tract elements regulate a cooperative mechanism for Triticum mosaic virus internal ribosomal entry site activity. Frontiers in Plant Science 13: 864832. 35498652 10.3389/fpls.2022.864832 PMC9042117

[nph70400-bib-0074] Jaramillo‐Mesa H , Gannon M , Holshbach E , Zhang J , Roberts R , Buettner M , Rakotondrafara AM . 2019. The Triticum mosaic virus internal ribosome entry site relies on a picornavirus‐like YX‐AUG motif to designate the preferred translation initiation site and to likely target the 18S rRNA. Journal of Virology 93: 10–1128. 10.1128/JVI.01705-18 PMC6384068 30541835

[nph70400-bib-0075] Jobling SA , Gehrke L . 1987. Enhanced translation of chimaeric messenger RNAs containing a plant viral untranslated leader sequence. Nature 325: 622–625. 3492677 10.1038/325622a0

[nph70400-bib-0076] Joshi CP , Zhou H , Huang X , Chiang VL . 1997. Context sequences of translation initiation codon in plants. Plant Molecular Biology 35: 993–1001. 9426620 10.1023/a:1005816823636

[nph70400-bib-0077] Jung H , Kim J‐K , Ha S‐H . 2011. Use of animal viral internal ribosome entry site sequence makes multiple truncated transcripts without mediating polycistronic expression in rice. Journal of Korean Society for Applied Biological Chemistry 54: 678–684.

[nph70400-bib-0078] Kamura N , Sawasaki T , Kasahara Y , Takai K , Endo Y . 2005. Selection of 5′‐untranslated sequences that enhance initiation of translation in a cell‐free protein synthesis system from wheat embryos. Bioorganic & Medicinal Chemistry Letters 15: 5402–5406. 16213724 10.1016/j.bmcl.2005.09.013

[nph70400-bib-0079] Kanoria S , Burma PK . 2012. A 28 nt long synthetic 5′ UTR (synJ) as an enhancer of transgene expression in dicotyledonous plants. BMC Biotechnology 12: 1–14. 23140609 10.1186/1472-6750-12-85 PMC3536603

[nph70400-bib-0080] Kehr J , Kragler F . 2018. Long distance RNA movement. New Phytologist 218: 29–40. 29418002 10.1111/nph.15025

[nph70400-bib-0081] Kertesz S , Kerenyi Z , Merai Z , Bartos I , Palfy T , Barta E , Silhavy D . 2006. Both introns and long 3′‐UTRs operate as *cis*‐acting elements to trigger nonsense‐mediated decay in plants. Nucleic Acids Research 34: 6147–6157. 17088291 10.1093/nar/gkl737 PMC1693880

[nph70400-bib-0082] Kneller ELP , Rakotondrafara AM , Miller WA . 2006. Cap‐independent translation of plant viral RNAs. Virus Research 119: 63–75. 16360925 10.1016/j.virusres.2005.10.010 PMC1880899

[nph70400-bib-0083] Kozak M . 1981. Possible role of flanking nucleotides in recognition of the AUG initiator codon by eukaryotic ribosomes. Nucleic Acids Research 9: 5233–5252. 7301588 10.1093/nar/9.20.5233 PMC327517

[nph70400-bib-0084] Kozak M . 1986. Point mutations define a sequence flanking the AUG initiator codon that modulates translation by eukaryotic ribosomes. Cell 44: 283–292. 3943125 10.1016/0092-8674(86)90762-2

[nph70400-bib-0085] Kozak M . 1987a. An analysis of 5′‐noncoding sequences from 699 vertebrate messenger RNAs. Nucleic Acids Research 15: 8125–8148. 3313277 10.1093/nar/15.20.8125 PMC306349

[nph70400-bib-0086] Kozak M . 1987b. Effects of intercistronic length on the efficiency of reinitiation by eucaryotic ribosomes. Molecular and Cellular Biology 7: 3438–3445. 3683388 10.1128/mcb.7.10.3438-3445.1987 PMC367994

[nph70400-bib-0087] Kozak M . 1989. Circumstances and mechanisms of inhibition of translation by secondary structure in Eucaryotic mRNAs. Molecular and Cellular Biology 9: 5134–5142. 2601712 10.1128/mcb.9.11.5134-5142.1989 PMC363665

[nph70400-bib-0088] Kozak M . 1990. Downstream secondary structure facilitates recognition of initiator codons by eukaryotic ribosomes. Proceedings of the National Academy of Sciences, USA 87: 8301–8305. 10.1073/pnas.87.21.8301 PMC54943 2236042

[nph70400-bib-0089] Kozak M . 2001. Constraints on reinitiation of translation in mammals. Nucleic Acids Research 29: 5226–5232. 11812856 10.1093/nar/29.24.5226 PMC97554

[nph70400-bib-0090] Kumar D , An C‐I , Yokobayashi Y . 2009. Conditional RNA interference mediated by allosteric ribozyme. Journal of the American Chemical Society 131: 13906–13907. 19788322 10.1021/ja905596t

[nph70400-bib-0091] Kwok CK , Ding Y , Shahid S , Assmann SM , Bevilacqua PC . 2015. A stable RNA g‐quadruplex within the 5′‐UTR of *Arabidopsis thaliana* ATR mRNA inhibits translation. Biochemical Journal 467: 91–102. 25793418 10.1042/BJ20141063

[nph70400-bib-0092] Kwon CS , Chung WI , Paek K‐H . 1997. Ribozyme mediated targeting of cucumber mosaic virus RNA 1 and 2 in transgenic tobacco plants. Molecules and Cells 7: 326–334. 9264018

[nph70400-bib-0093] Laing WA , Martínez‐Sánchez M , Wright MA , Bulley SM , Brewster D , Dare AP , Rassam M , Wang D , Storey R , Macknight RC *et al*. 2015. An upstream open reading frame is essential for feedback regulation of ascorbate biosynthesis in Arabidopsis. Plant Cell 27: 772–786. 25724639 10.1105/tpc.114.133777 PMC4558653

[nph70400-bib-0094] Larsen B , Hofmann R , Camacho IS , Clarke RW , Lagarias JC , Jones AR , Jones AM . 2023. Highlighter: an optogenetic system for high‐resolution gene expression control in plants. PLoS Biology 21: e3002303. 37733664 10.1371/journal.pbio.3002303 PMC10513317

[nph70400-bib-0095] Lastovka F , Peyret H , Thomas SE , Lomonossoff GP , Chung BY . 2024. Controlling heterologous protein synthesis through a plant RNA ThermoSwitch. bioRxiv. doi: 10.1101/2024.10.07.616989. PMC13353011 41906157

[nph70400-bib-0096] Leamy KA , Assmann SM , Mathews DH , Bevilacqua PC . 2016. Bridging the gap between *in vitro* and *in vivo* RNA folding. Quarterly Reviews of Biophysics 49: e10. 27658939 10.1017/S003358351600007X PMC5269127

[nph70400-bib-0097] Lee JH , Won HJ , Oh E‐S , Oh M‐H , Jung JH . 2020. Golden gate cloning‐compatible DNA replicon/2A‐mediated polycistronic vectors for plants. Frontiers in Plant Science 11: 559365. 33193484 10.3389/fpls.2020.559365 PMC7609577

[nph70400-bib-0098] Legen J , Ruf S , Kroop X , Wang G , Barkan A , Bock R , Schmitz‐Linneweber C . 2018. Stabilization and translation of synthetic operon‐derived mRNA s in chloroplasts by sequences representing PPR protein‐binding sites. The Plant Journal 94: 8–21. 29418028 10.1111/tpj.13863

[nph70400-bib-0099] Lin MT , Occhialini A , Andralojc PJ , Parry MA , Hanson MR . 2014. A faster rubisco with potential to increase photosynthesis in crops. Nature 513: 547–550. 25231869 10.1038/nature13776 PMC4176977

[nph70400-bib-0100] Lin Y , May GE , Kready H , Nazzaro L , Mao M , Spealman P , Creeger Y , McManus CJ . 2019. Impacts of uORF codon identity and position on translation regulation. Nucleic Acids Research 47: 9358–9367. 31392980 10.1093/nar/gkz681 PMC6755093

[nph70400-bib-0101] Lin Y‐H , Chang K‐Y . 2016. Rational design of a synthetic mammalian riboswitch as a ligand‐responsive‐1 ribosomal frame‐shifting stimulator. Nucleic Acids Research 44: 9005–9015. 27521370 10.1093/nar/gkw718 PMC5062990

[nph70400-bib-0102] Lomsadze A , Gemayel K , Tang S , Borodovsky M . 2018. Modeling leaderless transcription and atypical genes results in more accurate gene prediction in prokaryotes. Genome Research 28: 1079–1089. 29773659 10.1101/gr.230615.117 PMC6028130

[nph70400-bib-0103] Long BM , Hee WY , Sharwood RE , Rae BD , Kaines S , Lim Y‐L , Nguyen ND , Massey B , Bala S , von Caemmerer S *et al*. 2018. Carboxysome encapsulation of the CO_2_‐fixing enzyme rubisco in tobacco chloroplasts. Nature Communications 9: 1–14. 10.1038/s41467-018-06044-0 PMC6120970 30177711

[nph70400-bib-0104] Lu Y , Rijzaani H , Karcher D , Ruf S , Bock R . 2013. Efficient metabolic pathway engineering in transgenic tobacco and tomato plastids with synthetic multigene operons. Proceedings of the National Academy of Sciences, USA 110: E623–E632. 10.1073/pnas.1216898110 PMC3581966 23382222

[nph70400-bib-0105] Lukaszewicz M , Feuermann M , Jérouville B , Stas A , Boutry M . 2000. *In vivo* evaluation of the context sequence of the translation initiation codon in plants. Plant Science 154: 89–98. 10725562 10.1016/s0168-9452(00)00195-3

[nph70400-bib-0106] Luukkonen B , Tan W , Schwartz S . 1995. Efficiency of reinitiation of translation on human immunodeficiency virus type 1 mRNAs is determined by the length of the upstream open reading frame and by intercistronic distance. Journal of Virology 69: 4086–4094. 7769666 10.1128/jvi.69.7.4086-4094.1995 PMC189143

[nph70400-bib-0107] Macedo‐Osorio KS , Martínez‐Antonio A , Badillo‐Corona JA . 2021. Pas de trois: an overview of penta‐, tetra‐, and octo‐tricopeptide repeat proteins from *chlamydomonas reinhardtii* and their role in chloroplast gene expression. Frontiers in Plant Science 12: 775366. 34868174 10.3389/fpls.2021.775366 PMC8635915

[nph70400-bib-0108] Mahmudul H , AbrahamPaul E , MitchellJulie C , TuskanGerald A , EckertCarrie A , DoktyczMitchel J , TschaplinskiTimothy J *et al*. 2022. Biological and molecular components for genetically engineering biosensors in plants. Biodesign Research 2022: 9863496. 37850147 10.34133/2022/9863496 PMC10521658

[nph70400-bib-0109] Martin W , Kowallik KV . 1999. Annotated English translation of mereschkowsky's 1905 paper ‘Über natur und ursprung der chromatophoren im pflanzenreiche’. European Journal of Phycology 34: 287–295.

[nph70400-bib-0110] Martinez‐Salas E , Francisco‐Velilla R , Fernandez‐Chamorro J , Embarek AM . 2018. Insights into structural and mechanistic features of viral IRES elements. Frontiers in Microbiology 8: 2629. 29354113 10.3389/fmicb.2017.02629 PMC5759354

[nph70400-bib-0111] Matsuda D , Dreher TW . 2006. Close spacing of AUG initiation codons confers dicistronic character on a eukaryotic mRNA. RNA 12: 1338–1349. 16682564 10.1261/rna.67906 PMC1484435

[nph70400-bib-0112] Mayr C . 2017. Regulation by 3′‐untranslated regions. Annual Review of Genetics 51: 171–194. 10.1146/annurev-genet-120116-024704 28853924

[nph70400-bib-0113] Mehrshahi P , Nguyen GTD , Gorchs Rovira A , Sayer A , Llavero‐Pasquina M , Lim Huei Sin M , Medcalf EJ , Mendoza‐Ochoa GI , Scaife MA , Smith AG . 2020. Development of novel riboswitches for synthetic biology in the green alga chlamydomonas. ACS Synthetic Biology 9: 1406–1417. 32496044 10.1021/acssynbio.0c00082 PMC7309327

[nph70400-bib-0114] Merlo AO , Cowen N , Delate T , Edington B , Folkerts O , Hopkins N , Lemeiux C , Skokut T , Smith K , Woosley A *et al*. 1998. Ribozymes targeted to stearoyl–ACP 9 desaturase mRNA produce heritable increases of stearic acid in transgenic maize leaves. Plant Cell 10: 1603–1621. 9761789 10.1105/tpc.10.10.1603 PMC144353

[nph70400-bib-0115] Meshcheriakova YA , Saxena P , Lomonossoff GP . 2014. Fine‐tuning levels of heterologous gene expression in plants by orthogonal variation of the untranslated regions of a nonreplicating transient expression system. Plant Biotechnology Journal 12: 718–727. 24618146 10.1111/pbi.12175 PMC4265252

[nph70400-bib-0116] Meulewaeter F , Danthinne X , Va M , Montagu S , Cornelissen M . 1998. 5′‐and 3′‐sequences of satellite tobacco necrosis virus RNA promoting translation in tobacco. The Plant Journal 14: 169–176. 9628014 10.1046/j.1365-313x.1998.00104.x

[nph70400-bib-0117] Minskaia E , Nicholson J , Ryan MD . 2013. Optimisation of the foot‐and‐mouth disease virus 2A co‐expression system for biomedical applications. BMC Biotechnology 13: 1–11. 23968294 10.1186/1472-6750-13-67 PMC3765190

[nph70400-bib-0118] Miras M , Miller WA , Truniger V , Aranda MA . 2017. Non‐canonical translation in plant RNA viruses. Frontiers in Plant Science 8: 494. 28428795 10.3389/fpls.2017.00494 PMC5382211

[nph70400-bib-0119] Miras M , Sempere RN , Kraft JJ , Miller WA , Aranda MA , Truniger V . 2014. Interfamilial recombination between viruses led to acquisition of a novel translation‐enhancing RNA element that allows resistance breaking. New Phytologist 202: 233–246. 24372390 10.1111/nph.12650 PMC4337425

[nph70400-bib-0120] Moon K‐B , Jeon J‐H , Choi H , Park J‐S , Park S‐J , Lee H‐J , Park JM , Cho HS , Moon JS , Oh H *et al*. 2022. Construction of SARS‐CoV‐2 virus‐like particles in plant. Scientific Reports 12: 1005. 35046461 10.1038/s41598-022-04883-y PMC8770512

[nph70400-bib-0121] Moulin M , Nguyen GT , Scaife MA , Smith AG , Fitzpatrick TB . 2013. Analysis of chlamydomonas thiamin metabolism *in vivo* reveals riboswitch plasticity. Proceedings of the National Academy of Sciences, USA 110: 14622–14627. 10.1073/pnas.1307741110 PMC3767531 23959877

[nph70400-bib-0122] Mukhopadhyay J , Hausner G . 2021. Organellar introns in fungi, algae, and plants. Cells 10: 2001. 34440770 10.3390/cells10082001 PMC8393795

[nph70400-bib-0123] Mustoe AM , Brooks CL , Al‐Hashimi HM . 2014. Hierarchy of RNA functional dynamics. Annual Review of Biochemistry 83: 441–466. 10.1146/annurev-biochem-060713-035524 PMC4048628 24606137

[nph70400-bib-0124] Nakagawa S , Niimura Y , Gojobori T , Tanaka H , Miura K . 2008. Diversity of preferred nucleotide sequences around the translation initiation codon in eukaryote genomes. Nucleic Acids Research 36: 861–871. 18086709 10.1093/nar/gkm1102 PMC2241899

[nph70400-bib-0125] Nicholson BL , Wu B , Chevtchenko I , White KA . 2010. Tombusvirus recruitment of host translational machinery via the 3′ UTR. RNA 16: 1402–1419. 20507975 10.1261/rna.2135210 PMC2885689

[nph70400-bib-0126] Nicholson BL , Zaslaver O , Mayberry LK , Browning KS , White KA . 2013. Tombusvirus y‐shaped translational enhancer forms a complex with eIF4F and can be functionally replaced by heterologous translational enhancers. Journal of Virology 87: 1872–1883. 23192876 10.1128/JVI.02711-12 PMC3554133

[nph70400-bib-0127] Ogawa A . 2011. Rational design of artificial riboswitches based on ligand‐dependent modulation of internal ribosome entry in wheat germ extract and their applications as label‐free biosensors. RNA 17: 478–488. 21224378 10.1261/rna.2433111 PMC3039147

[nph70400-bib-0128] Ogawa A . 2013. Ligand‐dependent upregulation of ribosomal shunting. Chembiochem 14: 1539–1543. 23929633 10.1002/cbic.201300362

[nph70400-bib-0129] Ogawa A , Murashige Y , Tabuchi J , Omatsu T . 2017. Ligand‐responsive upregulation of 3′ CITE‐mediated translation in a wheat germ cell‐free expression system. Molecular BioSystems 13: 314–319. 27975086 10.1039/c6mb00748a

[nph70400-bib-0130] Ogawa A , Murashige Y , Takahashi H . 2018. Canonical translation‐modulating OFF‐riboswitches with a single aptamer binding to a small molecule that function in a higher eukaryotic cell‐free expression system. Bioorganic & Medicinal Chemistry Letters 28: 2353–2357. 29941191 10.1016/j.bmcl.2018.06.041

[nph70400-bib-0131] Ogawa A , Tabuchi J , Doi Y . 2014. Identification of short untranslated regions that sufficiently enhance translation in high‐quality wheat germ extract. Bioorganic & Medicinal Chemistry Letters 24: 3724–3727. 25037913 10.1016/j.bmcl.2014.07.004

[nph70400-bib-0132] Olins PO , Devine CS , Rangwala SH , Kavka KS . 1988. The T7 phage gene 10 leader RNA, a ribosome‐binding site that dramatically enhances the expression of foreign genes in *Escherichia coli* . Gene 73: 227–235. 3072257 10.1016/0378-1119(88)90329-0

[nph70400-bib-0133] Onishi M , Pringle JR . 2016. Robust transgene expression from bicistronic mRNA in the green alga *Chlamydomonas reinhardtii* . G3: Genes, Genomes, Genetics 6: 4115–4125. 27770025 10.1534/g3.116.033035 PMC5144980

[nph70400-bib-0134] Ossowski S , Schwab R , Weigel D . 2008. Gene silencing in plants using artificial microRNAs and other small RNAs. The Plant Journal 53: 674–690. 18269576 10.1111/j.1365-313X.2007.03328.x

[nph70400-bib-0135] Pajerowska‐Mukhtar KM , Wang W , Tada Y , Oka N , Tucker CL , Fonseca JP , Dong X . 2012. The HSF‐like transcription factor TBF1 is a major molecular switch for plant growth‐to‐defense transition. Current Biology 22: 103–112. 22244999 10.1016/j.cub.2011.12.015 PMC3298764

[nph70400-bib-0136] Palma M , Lejeune F . 2021. Deciphering the molecular mechanism of stop codon readthrough. Biological Reviews 96: 310–329. 33089614 10.1111/brv.12657

[nph70400-bib-0137] Park SV , Yang J‐S , Jo H , Kang B , Oh SS , Jung GY . 2019. Catalytic RNA, ribozyme, and its applications in synthetic biology. Biotechnology Advances 37: 107452. 31669138 10.1016/j.biotechadv.2019.107452

[nph70400-bib-0138] Peng H , Latifi B , Müller S , Lupták A , Chen IA . 2021. Self‐cleaving ribozymes: substrate specificity and synthetic biology applications. RSC Chemical Biology 2: 1370–1383. 34704043 10.1039/d0cb00207k PMC8495972

[nph70400-bib-0139] Peyret H , Brown JK , Lomonossoff GP . 2019. Improving plant transient expression through the rational design of synthetic 5′ and 3′ untranslated regions. Plant Methods 15: 1–13. 31548848 10.1186/s13007-019-0494-9 PMC6749642

[nph70400-bib-0140] Peyret H , Lomonossoff GP . 2015. When plant virology met agrobacterium: the rise of the deconstructed clones. Plant Biotechnology Journal 13: 1121–1135. 26073158 10.1111/pbi.12412 PMC4744784

[nph70400-bib-0141] Pfalz J , Bayraktar OA , Prikryl J , Barkan A . 2009. Site‐specific binding of a PPR protein defines and stabilizes 5′ and 3′ mRNA termini in chloroplasts. EMBO Journal 28: 2042–2052. 19424177 10.1038/emboj.2009.121 PMC2718276

[nph70400-bib-0142] Pooggin MM , Hohn T , Futterer J . 2000. Role of a short open reading frame in ribosome shunt on the cauliflower mosaic virus RNA leader. Journal of Biological Chemistry 275: 17288–17296. 10747993 10.1074/jbc.M001143200

[nph70400-bib-0143] Pöyry TA , Kaminski A , Jackson RJ . 2004. What determines whether mammalian ribosomes resume scanning after translation of a short upstream open reading frame? Genes & Development 18: 62–75. 14701882 10.1101/gad.276504 PMC314277

[nph70400-bib-0144] Prikryl J , Rojas M , Schuster G , Barkan A . 2011. Mechanism of RNA stabilization and translational activation by a pentatricopeptide repeat protein. Proceedings of the National Academy of Sciences, USA 108: 415–420. 10.1073/pnas.1012076108 PMC3017144 21173259

[nph70400-bib-0145] Pydiura M , Blume YB . 2023. Mechanisms of intron‐mediated enhancement of expression: welcome to the hotel California. Cytology and Genetics 57: 413–431.

[nph70400-bib-0146] Rakotondrafara AM , Polacek C , Harris E , Miller WA . 2006. Oscillating kissing stem–loop interactions mediate 5′ scanning‐dependent translation by a viral 3′‐cap‐independent translation element. RNA 12: 1893–1906. 16921068 10.1261/rna.115606 PMC1581982

[nph70400-bib-0147] Ramundo S , Rochaix J‐D . 2015. Controlling expression of genes in the unicellular alga *chlamydomonas reinhardtii* with a vitamin‐repressible riboswitch. Methods in Enzymology 2: 267–281. 10.1016/bs.mie.2014.10.035 25605390

[nph70400-bib-0148] Rangan L , Vogel C , Srivastava A . 2008. Analysis of context sequence surrounding translation initiation site from complete genome of model plants. Molecular Biotechnology 39: 207–213. 18236175 10.1007/s12033-008-9036-9

[nph70400-bib-0149] Roberts R , Mayberry LK , Browning KS , Rakotondrafara AM . 2017. The Triticum mosaic virus 5′ leader binds to both eIF4G and eIFiso4G for translation. PLoS ONE 12: e0169602. 28046134 10.1371/journal.pone.0169602 PMC5207729

[nph70400-bib-0150] Roberts R , Zhang J , Mayberry LK , Tatineni S , Browning KS , Rakotondrafara AM . 2015. A unique 5′ translation element discovered in Triticum mosaic virus. Journal of Virology 89: 12427–12440. 26423954 10.1128/JVI.02099-15 PMC4665250

[nph70400-bib-0151] Rojas M , Yu Q , Williams‐Carrier R , Maliga P , Barkan A . 2019. Engineered PPR proteins as inducible switches to activate the expression of chloroplast transgenes. Nature Plants 5: 505–511. 31036912 10.1038/s41477-019-0412-1

[nph70400-bib-0152] Ryan MD , King AM , Thomas GP . 1991. Cleavage of foot‐and‐mouth disease virus polyprotein is mediated by residues located within a 19 amino acid sequence. Journal of General Virology 72: 2727–2732. 1658199 10.1099/0022-1317-72-11-2727

[nph70400-bib-0153] Sahoo S , Singh D , Singh A , Pandit M , Vasu K , Som S , Pullagurla NJ , Laha D , Eswarappa SM . 2022. Identification and functional characterization of mRNAs that exhibit stop codon readthrough in *Arabidopsis thaliana* . Journal of Biological Chemistry 298: 102173. 35752360 10.1016/j.jbc.2022.102173 PMC9293766

[nph70400-bib-0154] Scharff LB , Childs L , Walther D , Bock R . 2011. Local absence of secondary structure permits translation of mRNAs that lack ribosome‐binding sites. PLoS Genetics 7: e1002155. 21731509 10.1371/journal.pgen.1002155 PMC3121790

[nph70400-bib-0155] Shanidze N , Lenkeit F , Hartig JS , Funck D . 2020. A theophylline‐responsive riboswitch regulates expression of nuclear‐encoded genes. Plant Physiology 182: 123–135. 31704721 10.1104/pp.19.00625 PMC6945857

[nph70400-bib-0156] Sharma P , Yan F , Doronina VA , Escuin‐Ordinas H , Ryan MD , Brown JD . 2012. 2A peptides provide distinct solutions to driving stop‐carry on translational recoding. Nucleic Acids Research 40: 3143–3151. 22140113 10.1093/nar/gkr1176 PMC3326317

[nph70400-bib-0157] Shen R , Yao Q , Zhong D , Zhang X , Li X , Cao X , Dong C , Tian Y , Zhu J‐K , Lu Y . 2023. Targeted insertion of regulatory elements enables translational enhancement in rice. Frontiers in Plant Science 14: 1134209. 37063194 10.3389/fpls.2023.1134209 PMC10102426

[nph70400-bib-0158] Shingu Y , Yokomizo S , Kimura M , Ono Y , Yamaguchi I , Hamamoto H . 2006. Conferring cadmium resistance to mature tobacco plants through metal‐adsorbing particles of tomato mosaic virus vector. Plant Biotechnology Journal 4: 281–288. 17147634 10.1111/j.1467-7652.2006.00180.x

[nph70400-bib-0159] Simon AE , Miller WA . 2013. 3′ cap‐independent translation enhancers of plant viruses. Annual Review of Microbiology 67: 21–42. 10.1146/annurev-micro-092412-155609 PMC4034384 23682606

[nph70400-bib-0160] Son S , Park SR . 2023. Plant translational reprogramming for stress resilience. Frontiers in Plant Science 14: 1151587. 36909402 10.3389/fpls.2023.1151587 PMC9998923

[nph70400-bib-0161] Staub JM , Maliga P . 1995. Expression of a chimeric uidA gene indicates that polycistronic mRNAs are efficiently translated in tobacco plastids. The Plant Journal 7: 845–848. 7773311 10.1046/j.1365-313x.1995.07050845.x

[nph70400-bib-0162] Stern DB , Gruissem W . 1987. Control of plastid gene expression: 3′ inverted repeats act as mRNA processing and stabilizing elements, but do not terminate transcription. Cell 51: 1145–1157. 3690662 10.1016/0092-8674(87)90600-3

[nph70400-bib-0163] Sugio T , Matsuura H , Matsui T , Matsunaga M , Nosho T , Kanaya S , Shinmyo A , Kato K . 2010. Effect of the sequence context of the AUG initiation codon on the rate of translation in dicotyledonous and monocotyledonous plant cells. Journal of Bioscience and Bioengineering 109: 170–173. 20129102 10.1016/j.jbiosc.2009.07.009

[nph70400-bib-0164] Tabuchi T , Okada T , Azuma T , Nanmori T , Yasuda T . 2006. Posttranscriptional regulation by the upstream open reading frame of the phosphoethanolamine n‐methyltransferase gene. Bioscience, Biotechnology, and Biochemistry 70: 2330–2334. 16960350 10.1271/bbb.60309

[nph70400-bib-0165] Tabuchi T , Yokobayashi Y . 2021. Cell‐free riboswitches. RSC Chemical Biology 2: 1430–1440. 34704047 10.1039/d1cb00138h PMC8496063

[nph70400-bib-0166] Tang J , Chen S , Jia G . 2023. Detection, regulation, and functions of RNA N6‐methyladenosine modification in plants. Plant Communications 4: 100546. 36627844 10.1016/j.xplc.2023.100546 PMC10203383

[nph70400-bib-0167] Tang J , Wang X . 2024. Strategies for RNA m6A modification application in crop improvement. Frontiers in Plant Science 15: 1477240. 39474221 10.3389/fpls.2024.1477240 PMC11518802

[nph70400-bib-0168] Thomas SE , Balcerowicz M , Chung BY‐W . 2022. RNA structure mediated thermoregulation: what can we learn from plants? Frontiers in Plant Science 13: 938570. 36092413 10.3389/fpls.2022.938570 PMC9450479

[nph70400-bib-0169] Tian L , Chou H‐L , Fukuda M , Kumamaru T , Okita TW . 2020. mRNA localization in plant cells. Plant Physiology 182: 97–109. 31611420 10.1104/pp.19.00972 PMC6945871

[nph70400-bib-0170] Tiwari M , Sharma D , Trivedi PK . 2014. Artificial microRNA mediated gene silencing in plants: progress and perspectives. Plant Molecular Biology 86: 1–18. 25022825 10.1007/s11103-014-0224-7

[nph70400-bib-0171] Truniger V , Miras M , Aranda MA . 2017. Structural and functional diversity of plant virus 3′‐cap‐independent translation enhancers (3′‐CITEs). Frontiers in Plant Science 8: 2047. 29238357 10.3389/fpls.2017.02047 PMC5712577

[nph70400-bib-0172] Urwin P , Yi L , Martin H , Atkinson H , Gilmartin PM . 2000. Functional characterization of the EMCV IRES in plants. The Plant Journal 24: 583–589. 11123797 10.1046/j.1365-313x.2000.00904.x

[nph70400-bib-0173] Valkov VT , Gargano D , Manna C , Formisano G , Dix PJ , Gray JC , Scotti N , Cardi T . 2011. High efficiency plastid transformation in potato and regulation of transgene expression in leaves and tubers by alternative 5′ and 3′ regulatory sequences. Transgenic Research 20: 137–151. 20464632 10.1007/s11248-010-9402-9

[nph70400-bib-0174] Verhounig A , Karcher D , Bock R . 2010. Inducible gene expression from the plastid genome by a synthetic riboswitch. Proceedings of the National Academy of Sciences, USA 107: 6204–6209. 10.1073/pnas.0914423107 PMC2852001 20308585

[nph70400-bib-0175] Verma K , Modgil M . 2024. RNA interference (RNAi) mediated technique for combating plant diseases: Harnessing nanoparticles for effective delivery and enhanced efficacy. Plant Cell, Tissue and Organ Culture 157: 53.

[nph70400-bib-0176] Von Arnim AG , Jia Q , Vaughn JN . 2014. Regulation of plant translation by upstream open reading frames. Plant Science 214: 1–12. 24268158 10.1016/j.plantsci.2013.09.006

[nph70400-bib-0177] Wachter A , Tunc‐Ozdemir M , Grove BC , Green PJ , Shintani DK , Breaker RR . 2007. Riboswitch control of gene expression in plants by splicing and alternative 3′ end processing of mRNAs. Plant Cell 19: 3437–3450. 17993623 10.1105/tpc.107.053645 PMC2174889

[nph70400-bib-0178] Wang H , Zhang D , Chen M , Meng X , Bai S , Xin P , Yan J , Chu J , Li J , Yu H . 2024. Genome editing of 3′ UTR‐embedded inhibitory region enables generation of gene knock‐up alleles in plants. Plant Communications 5: 568. 10.1016/j.xplc.2023.100745 PMC10943523 37946411

[nph70400-bib-0179] Wang J , Shin B‐S , Alvarado C , Kim J‐R , Bohlen J , Dever TE , Puglisi JD . 2022. Rapid 40S scanning and its regulation by mRNA structure during eukaryotic translation initiation. Cell 185: 4474–4487. 36334590 10.1016/j.cell.2022.10.005 PMC9691599

[nph70400-bib-0180] Wang X , Marchisio MA . 2021. Synthetic polycistronic sequences in eukaryotes. Synthetic and Systems Biotechnology 6: 254–261. 34584993 10.1016/j.synbio.2021.09.003 PMC8449083

[nph70400-bib-0181] Wang Z , Treder K , Miller WA . 2009. Structure of a viral cap‐independent translation element that functions via high affinity binding to the eIF4E subunit of eIF4F. Journal of Biological Chemistry 284: 14189–14202. 19276085 10.1074/jbc.M808841200 PMC2682867

[nph70400-bib-0182] Weiner I , Shahar N , Marco P , Yacoby I , Tuller T . 2019. Solving the riddle of the evolution of shine‐dalgarno based translation in chloroplasts. Molecular Biology and Evolution 36: 2854–2860. 31503284 10.1093/molbev/msz210

[nph70400-bib-0183] Wells DR , Tanguay RL , Le H , Gallie DR . 1998. HSP101 functions as a specific translational regulatory protein whose activity is regulated by nutrient status. Genes & Development 12: 3236–3251. 9784498 10.1101/gad.12.20.3236 PMC317219

[nph70400-bib-0184] Wen J‐D , Kuo S‐T , Chou H‐HD . 2021. The diversity of shine‐dalgarno sequences sheds light on the evolution of translation initiation. RNA Biology 18: 1489–1500. 33349119 10.1080/15476286.2020.1861406 PMC8583243

[nph70400-bib-0185] Wiese A , Elzinga N , Wobbes B , Smeekens S . 2004. A conserved upstream open reading frame mediates sucrose‐induced repression of translation. Plant Cell 16: 1717–1729. 15208401 10.1105/tpc.019349 PMC514156

[nph70400-bib-0186] Win MN , Smolke CD . 2007. A modular and extensible RNA‐based gene‐regulatory platform for engineering cellular function. Proceedings of the National Academy of Sciences, USA 104: 14283–14288. 10.1073/pnas.0703961104 PMC1964840 17709748

[nph70400-bib-0187] Win MN , Smolke CD . 2008. Higher‐order cellular information processing with synthetic RNA devices. Science 322: 456–460. 18927397 10.1126/science.1160311 PMC2805114

[nph70400-bib-0188] Wu S , Li X , Wang G . 2022. tRNA‐like structures and their functions. The FEBS Journal 289: 5089–5099. 34117728 10.1111/febs.16070

[nph70400-bib-0189] Wurbs D , Ruf S , Bock R . 2007. Contained metabolic engineering in tomatoes by expression of carotenoid biosynthesis genes from the plastid genome. The Plant Journal 49: 276–288. 17241450 10.1111/j.1365-313X.2006.02960.x

[nph70400-bib-0190] Xie J , Zhuang Z , Gou S , Zhang Q , Wang X , Lan T , Lian M , Li N , Liang Y , Ouyang Z *et al*. 2023. Precise genome editing of the kozak sequence enables bidirectional and quantitative modulation of protein translation to anticipated levels without affecting transcription. Nucleic Acids Research 51: 10075–10093. 37650635 10.1093/nar/gkad687 PMC10570039

[nph70400-bib-0191] Xing S , Chen K , Zhu H , Zhang R , Zhang H , Li B , Gao C . 2020. Fine‐tuning sugar content in strawberry. Genome Biology 21: 1–14. 10.1186/s13059-020-02146-5 PMC7470447 32883370

[nph70400-bib-0192] Xue C , Qiu F , Wang Y , Li B , Zhao KT , Chen K , Gao C . 2023. Tuning plant phenotypes by precise, graded downregulation of gene expression. Nature Biotechnology 41: 1758–1764. 10.1038/s41587-023-01707-w 36894598

[nph70400-bib-0193] Yang X , Cheema J , Zhang Y , Deng H , Duncan S , Umar MI , Zhao J , Liu Q , Cao X , Kwok CK *et al*. 2020. RNA g‐quadruplex structures exist and function *in vivo* in plants. Genome Biology 21: 1–23. 10.1186/s13059-020-02142-9 PMC7466424 32873317

[nph70400-bib-0194] Yang X , Yie Y , Zhu F , Liu Y , Kang L , Wang X , Tien P . 1997. Ribozyme‐mediated high resistance against potato spindle tuber viroid in transgenic potatoes. Proceedings of the National Academy of Sciences, USA 94: 4861–4865. 10.1073/pnas.94.10.4861 PMC24596 9144155

[nph70400-bib-0195] Yang X , Yu H , Duncan S , Zhang Y , Cheema J , Liu H , Benjamin Miller J , Zhang J , Kwok CK , Zhang H *et al*. 2022. RNA g‐quadruplex structure contributes to cold adaptation in plants. Nature Communications 13: 6224. 10.1038/s41467-022-34040-y PMC9585020 36266343

[nph70400-bib-0196] Yu Q , Barkan A , Maliga P . 2019. Engineered RNA‐binding protein for transgene activation in non‐green plastids. Nature Plants 5: 486–490. 31036913 10.1038/s41477-019-0413-0

[nph70400-bib-0197] Yu Q , Tungsuchat‐Huang T , Verma K , Radler MR , Maliga P . 2020. Independent translation of ORFs in dicistronic operons, synthetic building blocks for polycistronic chloroplast gene expression. The Plant Journal 103: 2318–2329. 32497322 10.1111/tpj.14864

[nph70400-bib-0198] Zhang B , Rapolu M , Kumar S , Gupta M , Liang Z , Han Z , Williams P , Su WW . 2017. Coordinated protein co‐expression in plants by harnessing the synergy between an intein and a viral 2A peptide. Plant Biotechnology Journal 15: 718–728. 27879048 10.1111/pbi.12670 PMC5425387

[nph70400-bib-0199] Zhang H , Si X , Ji X , Fan R , Liu J , Chen K , Wang D , Gao C . 2018. Genome editing of upstream open reading frames enables translational control in plants. Nature Biotechnology 36: 894–898. 10.1038/nbt.4202 30080209

[nph70400-bib-0200] Zhang J , Ruf S , Hasse C , Childs L , Scharff LB , Bock R . 2012. Identification of *cis*‐elements conferring high levels of gene expression in non‐green plastids. The Plant Journal 72: 115–128. 22639905 10.1111/j.1365-313X.2012.05065.x

[nph70400-bib-0201] Zhang T , Li C , Zhu J , Li Y , Wang Z , Tong C‐Y , Xi Y , Han Y , Koiwa H , Peng X *et al*. 2024. Structured 3′ UTRs destabilize mRNAs in plants. Genome Biology 25: 54. 38388963 10.1186/s13059-024-03186-x PMC10885604

[nph70400-bib-0202] Zhang Y , Li H , Shen Y , Wang S , Tian L , Yin H , Shi J , Xing A , Zhang J , Ali U *et al*. 2024. Readthrough events in plants reveal plasticity of stop codons. Cell Reports 43: 12569. 10.1016/j.celrep.2024.113723 38300801

[nph70400-bib-0203] Zhong V , Archibald BN , Brophy JA . 2023. Transcriptional and post‐transcriptional controls for tuning gene expression in plants. Current Opinion in Plant Biology 71: 102315. 36462457 10.1016/j.pbi.2022.102315 PMC12061055

[nph70400-bib-0204] Zhou F , Karcher D , Bock R . 2007. Identification of a plastid intercistronic expression element (IEE) facilitating the expression of stable translatable monocistronic mRNAs from operons. The Plant Journal 52: 961–972. 17825052 10.1111/j.1365-313X.2007.03261.x PMC2230500

[nph70400-bib-0205] Zhou S , Chen M , Yuan Y , Xu Y , Pu Q , Ai X , Liu S , Du F , Huang X , Dong J *et al*. 2023. *Trans*‐acting aptazyme for conditional gene knockdown in eukaryotic cells. Molecular Therapy 33: 367–375. 37547296 10.1016/j.omtn.2023.07.014 PMC10400872

[nph70400-bib-0206] Zuo X , Wang J , Yu P , Eyler D , Xu H , Starich MR , Tiede DM , Simon AE , Kasprzak W , Schwieters CD *et al*. 2010. Solution structure of the cap‐independent translational enhancer and ribosome‐binding element in the 3′ UTR of turnip crinkle virus. Proceedings of the National Academy of Sciences, USA 107: 1385–1390. 10.1073/pnas.0908140107 PMC2803139 20080629

